# Immunogenicity and Safety of Reduced-Dose Intradermal vs Intramuscular Influenza Vaccines: A Systematic Review and Meta-analysis

**DOI:** 10.1001/jamanetworkopen.2020.35693

**Published:** 2021-02-09

**Authors:** Oluwaseun Egunsola, Fiona Clement, John Taplin, Liza Mastikhina, Joyce W. Li, Diane L. Lorenzetti, Laura E. Dowsett, Tom Noseworthy

**Affiliations:** 1 Department Community Health Sciences, University of Calgary Alberta, Canada; 2 Health Sciences Library, University of Calgary, Alberta, Canada

## Abstract

**Importance:**

Low-dose intradermal influenza vaccines could be a suitable alternative to full intramuscular dose during vaccine shortages.

**Objective:**

To compare the immunogenicity and safety of the influenza vaccine at reduced or full intradermal doses with full intramuscular doses to inform policy design in the event of vaccine shortages.

**Data Sources:**

MEDLINE, Embase, and the Cochrane Central Register of Controlled Trials were searched for studies published from 2010 until June 5, 2020.

**Study Selection:**

All comparative studies across all ages assessing the immunogenicity or safety of intradermal and intramuscular influenza vaccinations were included.

**Data Extraction and Synthesis:**

Data were extracted by a single reviewer and verified by a second reviewer. Discrepancies between reviewers were resolved through consensus. Random-effects meta-analysis was conducted.

**Main Outcomes and Measures:**

Primary outcomes included geometric mean titer, seroconversion, seroprotection, and adverse events.

**Results:**

A total of 30 relevant studies were included; 29 studies were randomized clinical trials with 13 759 total participants, and 1 study was a cohort study of 164 021 participants. There was no statistically significant difference in seroconversion rates between the 3-µg, 6-µg, 7.5-µg, and 9-µg intradermal vaccine doses and the 15-µg intramuscular vaccine dose for each of the H1N1, H3N2, and B strains, but rates were significantly higher with the 15-µg intradermal dose compared with the 15-µg intramuscular dose for the H1N1 strain (rate ratio [RR], 1.10; 95% CI, 1.01-1.20) and B strain (RR, 1.40; 95% CI, 1.13-1.73). Seroprotection rates for the 9-µg and 15-µg intradermal doses did not vary significantly compared with the 15-µg intramuscular dose for all the 3 strains, except for the 15-µg intradermal dose for the H1N1 strain, for which rates were significantly higher (RR, 1.05; 95% CI, 1.01-1.09). Local adverse events were significantly higher with intradermal doses than with the 15-µg intramuscular dose, particularly erythema (3-µg dose: RR, 9.62; 95% CI, 1.07-86.56; 6-µg dose: RR, 23.79; 95% CI, 14.42-39.23; 9-µg dose: RR, 4.56; 95% CI, 3.05-6.82; 15-µg dose: RR, 3.68; 95% CI, 3.19-4.25) and swelling (3-µg dose: RR, 20.16; 95% CI, 4.68-86.82; 9-µg dose: RR, 5.23; 95% CI, 3.58-7.62; 15-µg dose: RR, 3.47 ; 95% CI, 2.21-5.45). Fever and chills were significantly more common with the 9-µg intradermal dose than the 15-µg intramuscular dose (fever: RR, 1.36; 95% CI, 1.03-1.80; chills: RR, 1.24; 95% CI, 1.03-1.50) while all other systemic adverse events were not statistically significant for all other doses.

**Conclusions and Relevance:**

These findings suggest that reduced-dose intradermal influenza vaccination could be a reasonable alternative to standard dose intramuscular vaccination.

## Key Points

**Question:**

Is low-dose intradermal influenza vaccine a suitable alternative to regular dose intramuscular vaccine?

**Findings:**

In this systematic review and meta-analysis including 30 studies with a total of 177 780 participants, the seroconversion rates of low doses of intradermal influenza vaccine vs the 15-µg intramuscular dose for each of the H1N1, H3N2, and B strains were not statistically significantly different. Seroprotection rates for the 9-µg and 15-µg intradermal doses were not statistically significantly different from the 15-µg intramuscular dose, except for the 15-µg intradermal dose for the H1N1 strain, which was significantly higher.

**Meaning:**

These findings suggest that a low-dose intradermal influenza vaccine may be a suitable alternative to standard-dose intramuscular vaccine.

## Teaser

This systematic review and meta-analysis examines the safety and efficacy of reduced- or full-dose influenza vaccine administered intradermally vs standard-dose administered intramuscularly.

## Introduction

Influenza infection causes 3 to 5 million severe illnesses and approximately half a million annual deaths globally.^1^ It is a highly contagious disease characterized by high fever, cough, sore throat, headache, chills, lack of appetite, and fatigue.^2^ Vaccinations are essential for prevention of influenza and can be administered intradermally or intramuscularly.^3^

Interest in intradermal influenza vaccines has arisen because of a presumed dose-sparing potential. An intradermal dose-sparing effect has been used successfully for other vaccines, such as rabies.^4,5^ If confirmed, this may mitigate potential vaccine shortages, which could occur from unanticipated loss of expected supplies or from excessive demand owing to high rates of infection, such as during pandemics.^6^ With the approval of new intradermal vaccines,^2,7,8^ new delivery devices have become available, including minineedles, microneedles, patches, and disposable syringe jet injectors.^3,9^ The recent international focus on the development of vaccines for coronavirus disease 2019 highlights the need to better understand the safety and efficacy of various vaccine delivery methods and doses.

Intradermal vaccinations are believed to have a dose-sparing effect^3^; therefore, smaller doses of intradermal vaccines may be sufficient to produce an antigenic response that is similar to standard intramuscular doses. This is physiologically plausible because the dermis is rich in Langerhans cells, dendritic cells that are very potent antigen-presenting cells capable of eliciting both cell-mediated and humoral immune responses via antigen presentation to CD4^+^ and CD8^+^ T cells, and eventual B cell activation to produce high levels of antigen-specific antibodies. Intramuscular injection bypasses this dermal immune system response and delivers the vaccine directly into the muscular tissue, which has relatively few resident antigen-presenting cells.^10^

Previous studies have compared the immunogenicity and safety of intradermal and intramuscular influenza vaccines; however, the magnitude of the effect across all populations has not been recently examined. In this study, we synthesized the published literature on the immunogenicity and safety of the influenza vaccine at reduced or regular intradermal doses compared with a regular intramuscular dose.

## Methods

### Literature Search

A systematic review of the literature was completed. MEDLINE, Embase, and the Cochrane Central Register of Controlled Trials were searched for studies published from 2010 until June 5, 2020. Terms aimed at capturing the technology of interest, such as *intradermal*, *ID injection*, and *Mantoux* were combined using the Boolean Operator *and* with influenza terms. These terms were searched as keywords (title or abstract words) and as subject headings (eg, MEDLINE medical subject headings) as appropriate. The search excluded case reports, editorials, letters, and animal studies. The search strategy was developed by a research librarian and reviewed by another research librarian using the Peer Review of Electronic Search Strategies method^11^ (eAppendix in the Supplement). This search was supplemented by reviewing the reference lists of published systematic reviews, identified during the abstract screening, to ensure that all studies meeting the inclusion criteria were captured. This review follows the Preferred Reporting Items for Systematic Reviews and Meta-analyses (PRISMA) reporting guideline. This study is registered in the International Prospective Register of Systematic Reviews (PROSPERO), No. CRD42020190246.

### Literature Selection

Abstracts identified through database searching were screened by a single reviewer (O.E., J.T., or L.M.); all abstracts included at this stage proceeded to full-text review. Full-text publications were screened by a single reviewer (O.E., J.T., or L.M.). Calibration with a second reviewer (O.E., J.T., or L.M.) was completed prior to abstract screening and full-text review until greater than 70% agreement was reached. Publications were included if they met all the following inclusion criteria: comparative, including randomized and nonrandomized clinical trials, studies of the immunogenicity and safety of intradermal and intramuscular influenza vaccine involving participants of any age, published between 2010 and 2020. Non–English- or French-language studies, animal studies, studies involving patients who were immunocompromised, and studies with whole-virus vaccinations were excluded.

### Data Extraction

For all included studies, year of publication, country, study design, dates of recruitment, study inclusion and exclusion criteria, setting, patient characteristics, treatment protocol (eg, intention-to-treat, per-protocol), sample size, follow-up time, geometric mean titer (GMT, defined as the antilog of the arithmetic mean of the log-transformed antibody titers), seroconversion rate (percentage of participants with a 4-fold increase in hemagglutination inhibition [HAI] antibody titers) and seroprotection rates (the percentage of participants achieving an HAI titer ≥40), and all relevant outcomes were extracted by a single reviewer (O.E., J.T., or L.M.) and verified by a second reviewer (O.E. verified L.M., J.T. verified O.E., and L.M. verified J.T.) using standardized data extraction forms. Immunogenicity outcomes were extracted for only HAI assays. Discrepancies between reviewers during data extraction were resolved through consensus.

### Quality Assessment

The quality of randomized clinical trials was assessed using the Cochrane Handbook Risk of Bias Assessment Tool version 5.1.0.^12^ Each study was assessed using 5 criteria broadly covering the areas of randomization, deviation from intended intervention, missing outcome data, measurement of outcome, and selection of reporting result. Each criterion was assigned a rating of low, some, or high concerns.

The quality of observational studies was assessed using the Newcastle Ottawa Scale. Each study was assessed across 3 categories: selection, comparability, and outcome. Items within selection and comparability were assigned up to 1 star for high quality, while items within comparability were assigned a maximum of 2 stars, with a maximum total possible score of 9 stars.

Quality assessment was completed by a single reviewer and verified by a second reviewer (quality assessment by single reviewers and verified in pairs: O.E. verified L.M., J.T. verified O.E., and L.M. verified J.T.). Discrepancies were resolved through discussion. Studies were not excluded based on quality assessment.

### Statistical Analysis

Random-effects meta-analysis was conducted using the DerSimonian and Laird estimator^13^ for tau. Statistical heterogeneity was assessed using the *I*^2^ measure, with values greater or less than 50% considered high and low heterogeneity, respectively. A continuity correction of 0.5 was used, where appropriate, allowing the inclusion of zero-total event trials.^14^ Stratified analyses by dose were completed for the GMT, seroconversion, seroprotection, influenza or influenza-like illness, and adverse events. For dose stratification, different intradermal vaccine doses (3, 6, 7.5, 9 and 15 µg) were separately compared with a 15-µg intramuscular dose for each outcome. Only immunogenicity outcomes for days 21 through 30 after vaccination were analyzed. Subgroup analyses of immunogenicity outcomes were conducted for studies involving participants aged 60 years or older. Risk ratios (RRs) were calculated for categorical outcomes, and the ratio of geometric means calculated for GMT, as described by Friedrich el al.^15^ Publication bias for small studies with missing small effect sizes was assessed using an Egger test^16^ when the number of studies was greater than 4. When the Egger test was statistically significant (*P* < .05), the Duval-Tweedie trim-and-fill method^17^ was used to adjust for funnel plot asymmetry. All analyses were completed in R statistical software version 3.6.1 (R Project for Statistical Computing). P values were 2-sided, and statistical significance was set at *P* < .05. For RR comparisons, statistical significance was inferred from the 95% CIs, and actual *P* values were not generated. Data were analyzed from July 2 through 16, 2020.

## Results

### Study Characteristics

The search strategy yielded 914 unique citations, 245 of which were excluded after deduplication, and 624 were excluded after abstract review. A total of 45 studies proceeded to full-text review (Figure); of these, 15 studies were excluded for inappropriate study design for the aims of this review (5 studies), incorrect outcome (5 studies), duplicate publication (2 studies), incorrect study population (1 study), and publication year not of interest (1 study). A total of 30 relevant studies were included in the final data set (Figure).

**Figure.**
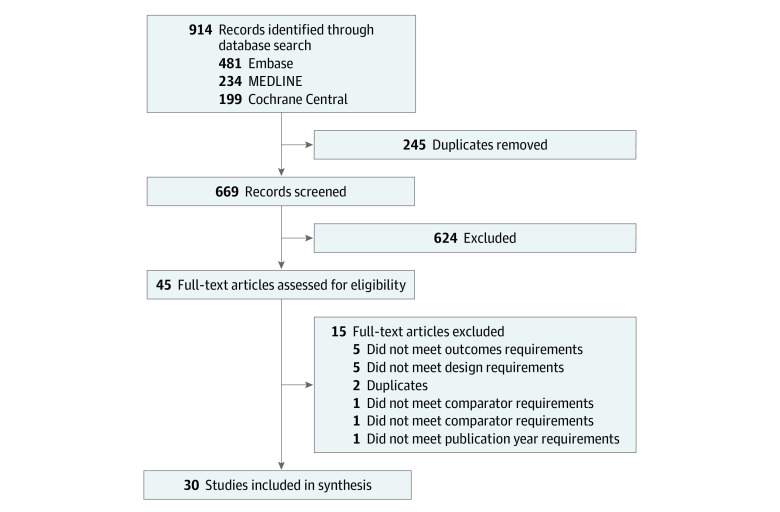
Flowchart of Included Studies

Of 30 included studies, 29 studies were randomized clinical trials with a total of 13 759 participants,^18,19,20,21,22,23,24,25,26,27,28,29,30,31,32,33,34,35,36,37,38,39,40,41,42,43,44,45,46^ and 1 study was a cohort study of 164 021 participants (Table 1).^47^ Sixteen studies were multi-center^18,19,20,22,23,29,31,33,34,37,38,41,43,44,47^; 12 studies were single-center^24,25,26,27,28,30,32,35,36,39,40,45^; and 2 studies did not report the setting.^21,42^ Approximately half of the studies (14 studies)^18,19,21,22,24,25,26,27,29,34,41,43,44,46^ involved only participants aged 60 years or older or reported data for participants aged 60 years or older.

**Table 1.** Characteristics of Included Studies

Source	Country	Participants		Dose, μg		Virus strains	Outcomes	Maximum follow-up, d
		No.	Age, range, y	ID	IM			
Ansaldi et al,^19^ 2013	Italy	47	≥60	15	15	A/H1N1 (A/California/7/2009, A/Genoa/1/11, A/Genoa/6/11, A/Genoa/24/11), A/H3N2 (A/Perth/16/2009), B Strain (B/Brisbane/60/2008)	AEs, GMT, MFI, Sc, Sp	90
Ansaldi et al,^18^ 2012	France, Belgium, Lithuania, Italy	50	≥60	15	15	A/H3N2 (Wisconsin/67/05, Genoa62/05, Genoa03/07, Brisbane/10/07, Genoa02/07,Genoa03/06)	GMT, MFI, Sc, Sp	21
Arnou et al,^20^ 2010	France, Italy, Belgium, Lithuania	1676	18-60	9	15	A/H1N1 (A/New Caledonia/20/99),A/H3N2(A/Wisconsin/67/2005), B Strain (B/Malaysia/2506/2004)	AEs, GMT, MFI, Sc, Sp	21
Boonnak et al,^21^ 2017	Thailand	221	60-88	15	15	A/H1N1(A/California/07/09), A/H3N2 (A/Songhka/308/13), B Strain (B/Phuket/287/13)	AEs, GMT, Sc, Sp	60
Camiloni et al,^22^ 2014	Italy	80	64−100	15	15	A/H1N1 (A/California/7/09,A/Perugia/06/12, A/Perugia/20/12, A/Perugia/44/12, A/Perugia/50/12), A/H3N2 (A/Perth/16/09), B Strain (B/Brisbane/60/08)	GMT, MFI, Sc, Sp	180
Carter et al,^23^ 2019	US	106	18-69	9	15	A/H1N1 (A/California/04/2009), A/H3N2 (A/Victoria/361/2011, A/Texas/50/2012), B Strain (B/Texas/6/2011,B/Wisconsin/1/2010, B/Massachusetts/2/2012)	AEs, GMT, Sc	21
Chan et al,^24^ 2014	Hong Kong	100	≥65	15	15	A/H1N1 (A/Victoria/361/2011), A/H3N2 (A/California/7/2009), B Strain (B/Massachusetts/2/2012)	AEs, GMT, MFI, Sc, Sp	180
Chi et al,^25^ 2010	US	130	≥65	9	15	A/H1N1 (A/Solomon Islands/3/2006), A/H3N2 (A/Wisconsin/67/2005), B Strain (B/Malaysia/2506/2004)	AEs, GMT, Sp	28
Chuaychoo et al,^26^ 2019	Thailand	80	≥60	9	15	A/H1N1 (A/California/7/2009), A/H3N2 (A/Perth/16/2009), B Strain (B/Brisbane/60/2008)	AEs, GMT, Sc, Sp	365
Chuaychoo et al,^27^ 2016	Thailand	149	60-94	9	15	A/H1N1 (A/California/7/2009), A/H3N2 (A/Perth/16/2009), B Strain (B/Brisbane/60/2008)	AEs, GMT, Sc, Sp, Influenza infection	28
Chuaychoo et al,^28^ 2010	Thailand	156	36-91	6	15	A/H1N1 (A/New Caledonia/20/99), A/H3N2 (A/California/7/2004), B Strain (B/Malaysia/2506/2004)	AEs, GMT, Sc, Sp	365
Della Cioppa et al,^29^ 2014	Germany, Poland, Belgium	257	≥65	6 or 12	15 or 30	A/H3N2 (A/Uruguay/716/2007)	AEs, GMT, MFI, Sc, Sp	22
Esposito et al,^30^ 2011	Italy	112	≥3	9 or 15	15	A/H1N1 (A/California/7/2009), A/H3N2 (A/Perth/16/2009), B Strain (B/Brisbane/60/2008)	AEs, GMT, MFI, Sc, Sp	28
Frenck et al,^31^ 2011	US	1571	18-64	3, 6, or 9	15	A/H1N1 (A/New Caledonia/20/99 IVR-116), A/H3N2 (A/Wyoming/03/2003 (an A/Fujian/411/2002-like strain), B Strain (B/Jiangsu/10/2003 (a B/Jiangsu/361/2002-like strain))	AEs, GMT, MFI, Sc, Sp	21
Garg et al,^32^ 2016	Thailand	80	18-60	15	15	A/H1N1, A/H3N2, B Strain	AEs, GMT, MFI, Sc, Sp	30
Gorse et al,^33^ 2013	US	3868	18-64	9	15	A/H1N1 (A/Brisbane/59/07), A/H3N2 (A/Uruguay/716/2007 X-175CA), B Strain (B/Florida/04/2006 Yamagata-like)	AEs, GMT, Sc, Sp	28
Han et al,^34^ 2013	South Korea	120	≥18	9 or 15	15	A/H1N1 (A/California/7/2009), A/H3N2 (A/Perth/16/2009), B Strain (B/Brisbane/60/2008)	AEs, GMT, Sc, Sp	21
Hung et al,^35^ 2016	China	160	18-30	15	15	A/H1N1 (A/California/07/2009, Prototype A/WSN/1933, A/HK/408027/09, A/H3N2 (A/Victoria/361/2011, A/HK/485197/14), B Strain (B/Massachusetts/2/2012), Others (B/HK/418078/11)	AEs, GMT, MFI, Sc, Sp	21
Hung et al,^37^ 2014	China	93	≥21	15	15	A/H1N1 (A/California/07/2009), A/H3N2 (A/Perth/16/2009), B Strain (B/Brisbane/60/2008)	AEs, GMT, MFI, Sc, Sp, Influenza	365
Hung et al,^36^ 2012	China	262	≥21	3 or 9	15	A/H1N1 (A/California/07/2009), A/H3N2 (A/Perth/16/2009), B Strain (B/Brisbane/60/2008)	AEs, GMT, MFI, Sc, Sp	21
Leung et al,^38^ 2017	US	336	18-64	NR	NR	A/H1N1 (A/California/07/2009), A/H3N2 (A/Perth/16/2009), B Strain (B/Brisbane/60/2008)	AEs, GMT, MFI, Sc, Sp	28
Levin et al,^46^ 2016	NR	370	≥65	7.5 or 15	15	A/H1N1 (A/California/07/2009), A/H3N2 (A/Victoria/361/2011), B Strain (B/Wisconsin/1/2010)	AE, Sc, Sp	90
Levin et al,^45^ 2014	Switzerland	280	18-60	3, 4.5, or 6	15	A/H1N1 (A/SolomonIslands/3/2006), A/H3N2 (A/Wisconsin/67/2005), B Strain (B/Malaysia/2506/2004)	AEs, GMT, MFI, Sc, Sp	21
Nougarede et al,^39^ 2014	France	80	18-40	9	15	A/H1N1 (A/SolomonIslands/3/2006), A/H3N2 (A/Wisconsin/67/2005), B Strain (B/Malaysia/2506/2004)	AEs, GMT, MFI, Sc, Sp, influenza-like illness	180
Patel et al,^40^ 2010	US	100	18-40	3 or 9	15	H5N1 (A/Vietnam/1203/2004)	AEs, GMT,Sc, Sp	28
Seo et al,^41^ 2014	South Korea	364	≥65	15	15	A/H1N1 (A/California/7/2009), A/H3N2 (A/Perth/16/2009), B Strain (B/Brisbane/60/2008)	AEs, GMT, MFI, Sc, Sp	180
Song et al,^42^ 2013	South Korea	96	18-30	3 or 7.5	15	A/H1N1 (A/New Caledonia/20/99), A/H3N2 (A/Wisconsin/67/2005), B Strain (B/Malaysia/2506/2004)	GMT, Sc, Sp	180
Tsang et al,^43^ 2014	US	1912	≥65	15 or 21	15	A/H1N1 (A/Solomon Islands/3/2006), A/H3N2 (A/Wisconsin/67/2005), B Strain (B/Malaysia/2506/2004)	AEs, GMT, MFI, Sc, Sp	28
Van Damme et al,^44^ 2010	France, Belgium	795	≥65	15	15	A/H1N1 (A/Solomon Islands/3/2006), A/H3N2 (/Wisconsin/67/2005), B Strain (B/Malaysia/2506/2004)	AEs, GMT, MFI, Sc, Sp	21
Puig Barbera et al,^47^ 2014^a^	Spain	164021	≥65	15	15	A/H1N1 (A/California/7/2009), A/H3N2 (A/Perth/16/2009), B Strain (B/Brisbane/60/2008)	Influenza	

Abbreviations: AE, adverse events; GMT, geometric mean titer; ID, intradermal; IM, intramuscular; NR, not reported; MFI, mean fold increase; Sc, seroconversion rate; Sp, seroprotection rate.

^a^ All other included studies were randomized clinical trials, but Puig Barbera et al^47^ was a cohort study.

### Quality Assessment

Most studies had bias stemming from the randomization process; 6 studies were at low risk of bias^20,23,24,30,32,35^; and 2 studies were at high risk.^28,34^ All but 2 low-risk studies^23,35^ had some risk of bias due to deviations from intended interventions. All included studies had low risk of bias due to missing outcome data. All but 1 high-risk study^34^ were of low risk of bias stemming from the measurement of outcomes. Lastly, all studies were of concern of bias regarding selection of the reported results. Overall, all studies except 2 high-risk studies,^28,34^ were of some concern for bias (eFigure 1 in the Supplement).

The only included cohort study was allocated 9 out of a possible 9 stars.^47^ It was judged to be representative of the exposed population. Exposure were ascertained from secure records, and outcomes were ascertained from record linkage. The cohorts were comparable, and follow-up was long and adequate.

### Meta-analyses

#### Seroconversion

Although there was high heterogeneity, no statistically significant difference in seroconversion rates was found between the 3-µg, 6-µg, 7.5-µg, and 9-µg intradermal vaccine doses vs the 15-µg intramuscular vaccine dose for each of the H1N1, H3N2, and B strains. The doses represent the amount of hemagglutinin present in each vaccine. Furthermore, the difference in the seroconversion rate for the H3N2 strain was also not statistically significant between the 15-µg intradermal dose and 15-µg intramuscular doses, but the seroconversion rate was significantly higher with the 15-µg intradermal dose compared with 15-µg intramuscular doses for the H1N1 strain (RR, 1.10; 95% CI, 1.01-1.20) and B strain (RR, 1.40; 95% CI, 1.13-1.73) (Table 2; eFigures 2-8 in the Supplement).

**Table 2.** Seroconversion, Seroprotection, and GMT of Intradermal Doses vs Standard 15-μg Intramuscular Dose of Influenza Vaccine

Intradermal dose	Studies, No.	Risk ratio (95% CI)	*I*^2^
Seroconversion			
H1N1			
3 µg	2	1.77 (0.43-7.28)	82.6
6 µg	3	1.00 (0.78-1.28)	87.7
7.5 µg	3	1.01 (0.80-1.28)	0
9 µg	10	1.02 (0.93-1.12)	59
15 µg	16	1.10 (1.01-1.20)^a^	50.5
H3N2			
3 µg	2	1.14 (0.56-2.31)	81.3
6 µg	3	0.98 (0.97-1.00)	0
7.5 µg	3	0.92 (0.63-1.33)	63.8
9 µg	11	1.01 (0.95-1.06)	38
15 µg	17	1.07 (0.99-1.17)	43.2
B Strain			
3 µg	2	1.46 (0.67-1.99)	53.5
6 µg	3	0.95 (0.68-1.32)	88.3
7.5 µg	3	1.21 (0.79-1.85)	43.9
9 µg	11	0.95 (0.84-1.08)	57.1
15 µg	16	1.40 (1.13-1.73)^a^	59.1
Seroprotection			
H1N1			
3 µg	3	1.00 (0.78-1.28)	87.7
6 µg	3	0.93 (0.88-0.99)^a^	37.5
7.5 µg	3	1.07 (1.01-1.12)^a^	0
9 µg	12	1.00 (0.98-1.03)	33
15 µg	17	1.05 (1.01-1.09)^a^	43.6
H3N2			
3 µg	3	0.98 (0.97-1.00)	0
6 µg	3	1.00 (0.99-1.01)	0
7.5 µg	3	1.01 (0.96-1.06)	36.6
9 µg	12	1.00 (0.99-1.00)	0
15 µg	18	1.01 (0.99-1.02)	25.9
B Strain			
3 µg	3	0.95 (0.68-1.32)	88.3
6 µg	3	0.92 (0.86-0.98)^a^	0
7.5 µg	3	1.13 (0.78-1.66)	58.2
9 µg	12	0.99 (0.95-1.03)	50
15 µg	16	1.03 (0.97-1.09)	48.5
GMT^b^			
H1N1			
3 µg	3	1.00 (0.54-1.84)	99.9
6 µg	2	0.88 (0.85-0.90)^a^	65.1
9 µg	11	1.04 (0.99-1.10)	99.8
15 µg	11	1.17 (0.95-1.42)	99.9
H3N2			
3 µg	3	0.90 (0.68-1.18)	99.4
6 µg	3	1.09 (0.90-1.32)	99
9 µg	11	1.08 (1.05-1.12)^a^	99.4
15 µg	11	1.16 (0.96-1.41)	100
B strain			
3 µg	3	0.80 (0.46-1.38)	99.9
6 µg	2	0.82 (0.67-1.01)	98.9
9 µg	11	0.93 (0.86-1.01)	99.9
15 µg	11	1.21 (1.11-1.32)^a^	99.8

Abbreviation: GMT, geometric mean titer.

^a^ *P* < 05.

^b^ Data are expressed as ratio of means for GMT.

#### Seroprotection

Seroprotection rates were significantly lower with the 6-µg intradermal dose vs the 15-μg intramuscular dose for the H1N1 strain (RR, 0.93; 95% CI, 0.88-0.99) and B strain (RR, 0.92; 95% CI, 0.86-0.98). For the 9-µg intradermal doses, seroprotection rates were not statistically significant compared with the 15-µg intramuscular dose for all 3 strains. The seroprotection rates for 15-µg intradermal and 15-µg intramuscular doses were also not statistically significantly different for H3N2 and B strains; however, the seroprotection rate for intradermal doses was significantly higher for the H1N1 strain compared with the 15-μg intramuscular dose (RR, 1.05; 95% CI,1.01-1.09) (Table 2; eFigures 9-15 in the Supplement).

### GMT

Although there was high heterogeneity, the GMTs were not statistically significantly different between the 3-µg and 6-µg intradermal doses and the 15-µg intramuscular dose for the 3 strains, except for a significant decrease for H1N1 observed with the 6-µg intradermal dose (RR, 0.88; 95% CI, 0.85-0.90). Similarly, GMTs were not statistically significant for the H1N1 and B strains when the 9-µg intradermal doses were compared with the 15-µg intramuscular dose, but GMT was significantly higher for the 9-µg intradermal dose of the H3N2 strain (RR, 1.08; 95% CI, 1.05-1.12). The 15-µg intradermal dose showed no statistically significant difference with the 15-µg intramuscular dose for the H1N1 and the H3N2 strains. However, the 15-µg intradermal dose was associated with significantly higher GMT for the B strain (RR, 1.21; 95% CI, 1.11-1.32) (Table 2; eFigures 16-21 in the Supplement).

### Immunogenicity in Older Adults

Subgroup analyses for immunogenicity in adults aged 60 years and older did not show statistically significant difference between the 9-µg intradermal dose and the 15-µg intramuscular doses, with respect to seroconversion, seroprotection, or GMT for each of the 3 strains. There was high heterogeneity among the studies. Seroprotection rates did not differ significantly between the 15-µg intradermal dose vs 15-µg intramuscular dose for the 3 strains, while seroconversion rate was significantly higher with the 15-µg intradermal dose compared with the 15-µg intramuscular dose for the B strain (RR, 1.41; 95% CI, 1.13- 1.75), as was GMT (RR, 1.19; 95% CI, 1.09-1.30) (Table 3).

**Table 3.** Immunogenicity of Intradermal Doses vs Standard 15-µg Intramuscular Dose of Influenza Vaccine Among Participants ≥60 Years

Intradermal dose	Pooled studies, No.	Risk ratio (95% CI)	*I*^2^
Seroconversion			
H1N1			
9 µg	2	1.01 (0.58-1.77)	87
15 µg	13	1.11 (1.00-1.24)	57.3
H3N2			
9 µg	2	1.02 (0.83-1.25)	0
15 µg	14	1.12 (1.00-1.25)	52.1
B strain			
9 µg	2	1.00 (0.60-1.67)	0
15 µg	13	1.41 (1.13-1.75)^a^	59
Seroprotection			
H1N1			
9 µg	4	0.98 (0.88-1.09)	24.1
15 µg	14	1.04 (1.00-1.09)	55.1
H3N2			
9 µg	4	1.03 (0.94-1.12)	0
15 µg	11	1.01 (0.99-1.03)	38.6
B strain			
9 µg	4	0.95 (0.71-1.27)	0
15 µg	12	1.03 (0.97-1.09)	45.3
GMT			
H1N1			
9 µg	4	0.96 (0.75-1.23)^b^	99.3
15 µg	11	1.11 (0.89-1.39)^b^	100
H3N2			
9 µg	4	1.07 (0.80-1.44)^b^	99.5
15 µg	7	1.13 (0.92-1.40)^b^	100
B strain			
9 µg	4	0.93 (0.72-1.20)^b^	99.5
15 µg	9	1.19 (1.09-1.30)^a^^,^^b^	99.8

Abbreviation: GMT, geometric mean titer.

^a^ *P* < .05.

^b^ Data are expressed as ratio of means for GMT.

### Influenza Infection or Influenza-Like Illness

A meta-analysis of 4 studies reporting clinical outcomes showed that the risk of influenza or influenza-like illness was significantly lower with intradermal vaccines compared with intramuscular vaccines (RR, 0.62; 95% CI, 0.49-0.77).^27,37,39,47^ However, there was no significant difference between the 2 routes of administration at intradermal dosages of 9 µg (RR, 0.61; 95% CI, 0.19-1.91)^27,39^ or 15 µg (RR, 0.68; 95% CI, 0.43-1.08).^37,47^

### Adverse Events

Local adverse events, including erythema, swelling, induration, pruritus, and ecchymosis, were significantly higher across the dose spectrum of intradermal vaccines compared with the standard intramuscular dose, particularly erythema (3-µg dose: RR, 9.62; 95% CI, 1.07-86.56; 6-µg dose: RR, 23.79; 95% CI, 14.42-39.23; 9-µg dose: RR, 4.56; 95% CI, 3.05-6.82; 15-µg dose: RR, 3.68; 95% CI, 3.19-4.25) and swelling (3-µg dose: RR, 20.16; 95% CI, 4.68-86.82; 9-µg dose: RR, 5.23; 95% CI, 3.58-7.62; 15-µg dose: RR, 3.47 ; 95% CI, 2.21-5.45). There was high heterogeneity among the pooled studies. Pain did not differ significantly between the 6-µg, 9-µg, or 15-µg intradermal doses vs the 15-µg intramuscular dose but was significantly lower with the 3-µg intradermal dose (Table 4; eFigures 22-25 in the Supplement). Differences in systemic adverse events, including headache, fever, malaise, arthralgia, myalgia, and nausea, were not statistically significant between the low intradermal doses and the standard intramuscular dose, and fever (RR, 1.36; 95% CI, 1.03-1.80) and chills (RR, 1.24; 95% CI, 1.03-1.50) were more common with the 9-µg intradermal dose than 15-µg intramuscular dose (Table 4; eFigures 26-29 in the Supplement).

**Table 4.** Local and Systemic Adverse Events Risks With Intradermal Doses vs 15 µg Intramuscular Dose of Influenza Vaccine

Intradermal dose	Pooled studies, No.	Risk ratio (95% CI)	*I*^2^
**Local adverse events**			
Ecchymosis			
9 µg	7	1.67 (1.12-2.48)^a^	55
15 µg	9	1.06 (0.73-1.57)	0
Erythema			
3 µg	3	9.62 (1.07-86.56)^a^	97.2
6 µg	2	23.79 (14.42-39.23)^a^	0
9 µg	14	4.56 (3.05-6.82)^a^	93.9
15 µg	16	3.68 (3.19-4.25)^a^	8.8
Induration			
9 µg	5	3.27 (1.65-6.46)^a^	95.4
15 µg	9	2.98 (2.32-3.84)^a^	42.6
Pain			
3 µg	4	0.34 (0.20-0.56)^a^	21.9
6 µg	2	0.98 (0.38-2.49)	68.3
9 µg	12	0.95 (0.86-1.05)	34.4
15 µg	16	0.94 (0.72-1.21)	61.3
Pruritus			
6 µg	2	15.22 (4.77-48.54)^a^	0
9 µg	9	4.24 (3.16-5.70)^a^	56.2
15 µg	6	4.01 (3.13-5.15)^a^	0
Swelling			
3 µg	2	20.16 (4.68-86.82)^a^	51.3
9 µg	13	5.23 (3.58-7.62)^a^	84.4
15 µg	12	3.47 (2.21-5.45)^a^	71.9
**Systemic adverse events**			
Arthralgia			
15 µg	3	1.17 (0.39-3.53)	22.7
Chills and shivering			
9 µg	7	1.24 (1.03-1.50)^a^	0
15 µg	10	1.08 (0.78-1.51)	0
Fever			
6 µg	2	0.54 (0.17-1.71)	34.5
9 µg	11	1.36 (1.03-1.80)^a^	0
15 µg	13	0.89 (0.59-1.34)	0
Headache			
3 µg	2	1.09 (0.86-1.37)	0
6 µg	2	0.83 (0.39-1.78)	68
9 µg	13	1.03 (0.96-1.11)	0
15 µg	9	1.16 (0.94-1.45)	0
Malaise			
9 µg	7	1.05 (0.94-1.20)	7.1
15 µg	14	0.97 (0.78-1.22)	0
Myalgia			
9 µg	12	1.24 (0.93-1.65)	74.8
15 µg	9	0.84 (0.63-1.12)	29.4
Nausea			
9 µg	3	0.93 (0.37-2.31)	0
15 µg	2	1.05 (0.33-3.33)	0

^a^ *P* < .05.

### Publication Bias

The Egger test for publication bias was statistically significant for the 15-µg intradermal and intramuscular doses comparison for the B strain seroconversion rate (intercept: 0.97; 95% CI, 0.21-1.73, *P* = .02) and the H3N2 strain seroprotection rate (intercept: 1.80; 95% CI, 0.43-3.17, *P* = .02). Bias correction using the trim-and-fill method did not change the statistical significance of the unadjusted results (eFigures 30-35 in the Supplement).

## Discussion

This systematic review and meta-analysis found that immunogenicity resulting from 3-µg, 6-µg, 7.5-µg and 9-µg influenza intradermal vaccination doses was not significantly different from full-dose 15-µg intramuscular vaccination for most viral strains, irrespective of patient age. However, the 15-µg intradermal vaccine showed significantly better immunogenicity for some of the outcomes and strains, suggesting that the immunological response may be dose-related. The risk of local adverse events, such as erythema, induration, swelling, and ecchymosis, was reduced with intramuscular vaccination; however, the risk of pain did not differ significantly between the 2 administration methods, with the exception of the 3-µg intradermal dose, which significantly lowered the risk of pain. The risks of systemic adverse events, such as headache, malaise, myalgia, and arthralgia, were similar with both administration methods.

The findings of this study are similar to those by Marra et al^48^ and 2 studies by Pileggi et al,^49,50^ which found no statistically significant difference between the different intradermal influenza vaccine doses and the 15-µg intramuscular influenza vaccine dose. It should be noted that Pileggi et al included studies involving only participants who were immunocompromised in one of their studies^49^ and only older adults in another.^50^ However, our systematic review excluded participants who were immunocompromised and carried out sensitivity analysis of studies involving older adults, given that old age^51^ and immunocompromise^52^ are known to attenuate immunological response. Although local skin reactions were more common with intradermal vaccinations, these reactions are generally well-accepted by vaccinees,^53,54^ who also find the microinjection systems to be more tolerable than the regular needles.^54^ These reactions are generally transient, with comparable rates of pain as intramuscular vaccination.^55^ Furthermore, the development of novel intradermal vaccine delivery systems, such as self-administrable patches with coated microprojections or biodegradable needles, could potentially improve vaccine acceptance and uptake.^56^ None of the studies in our review reported the use of nonneedle delivery systems. Intradermal administration requires advanced technical skill and special needles that present feasibility barriers to implementation. In Canada, an intradermal influenza vaccine is available off-label; however, most pharmacists are not licensed to administer intradermally despite administering approximately 30% of influenza doses every year.^57^

The results for immunogenicity and safety outcomes for this systematic review and meta-analysis were derived from only randomized clinical trials. This suggests a high level of evidence for these outcomes. The cohort study data were only included in the meta-analysis for influenza or influenza-like illness.

### Limitations

This study has some limitations. One limitation was the heterogeneity among the included studies, particularly with respect to the GMT outcome. This may be associated with the variation in the characteristics of the study participants, including age and comorbidities. However, heterogeneity persisted after stratifying the meta-analyses by age group. Other possible causes of heterogeneity include variations in vaccine factors, such as the use of adjuvants and differences in vaccine brands and delivery systems. Additionally, although the DerSimonian and Laird estimator of the between-study variance used in this study is the most commonly used method,^58^ it tends to produce narrower CIs, which may be less conservative in the representation of uncertainty in the estimation of between-study heterogeneity, especially when the number of studies included in the meta-analysis is small.^58,59^

## Conclusions

The findings of this systematic review and meta-analysis suggest that given the similarity in immunogenicity between the reduced dose intradermal and full dose intramuscular influenza vaccine, low-dose intradermal vaccine could be a reasonable alternative to standard-dose intramuscular vaccination. It will be important to determine if this dose-sparing finding holds true across age groups and for newer vaccines, particularly when recent high-dose formulations have demonstrated improved immunogenicity in older adults in whom immune responses have historically struggled.

## Notes

Supplement. **eAppendix.** Search Strategy **eFigure 1.** Quality Assessment of Randomized Clinical Trials **eFigure 2.** Seroconversion: 3 μg ID vs 15 μg IM **eFigure 3.** Seroconversion: 6 μg ID vs 15 μg IM **eFigure 4.** Seroconversion: 7.5 μg ID vs 15 μg IM **eFigure 5.** Seroconversion: 9 μg ID vs 15 μg IM in All Population **eFigure 6.** Seroconversion: in Older Adults 9 μg ID vs 15 μg IM **eFigure 7.** Seroconversion: 15 μg ID vs 15 μg IM **eFigure 8.** Seroconversion: in Older Adults 15 μg ID vs 15 μg IM **eFigure 9.** Seroprotection: 3 μg ID vs 15 μg IM **eFigure 10.** Seroprotection: 6 μg ID vs 15 μg IM **eFigure 11.** Seroprotection: 7.5 μg ID vs 15 μg IM **eFigure 12.** Seroprotection: 9 μg ID vs 15 μg IM **eFigure 13.** Seroprotection Older Adults: 9 μg ID vs 15 μg IM **eFigure 14.** Seroprotection: 15 μg ID vs 15 μg IM **eFigure 15.** Seroprotection in Older Adults: 15 μg ID vs 15 μg IM **eFigure 16.** GMT: 3 μg ID vs 15 μg IM **eFigure 17.** GMT: 6 μg ID vs 15 μg IM **eFigure 18.** GMT: 9 μg ID vs 15 μg IM **eFigure 19.** GMT: in Older Adults 9 μg ID vs 15 μg IM **eFigure 20.** GMT: 15 μg ID vs 15 μg IM **eFigure 21.** GMT in Older Adults: 15 μg ID vs 15 μg IM **eFigure 22.** Local Adverse Events: 3 μg ID vs 15 μg IM **eFigure 23.** Local Adverse Events: 6 μg ID vs 15 μg IM **eFigure 24.** Local Adverse Events: 9 μg ID vs 15 μg IM **eFigure 25.** Local Adverse Events: 15 μg ID vs 15 μg IM **eFigure 26.** Systemic Adverse Events: 3 μg ID vs 15 μg IM **eFigure 27.** Systemic Adverse Events: 6 μg ID vs 15 μg IM **eFigure 28.** Systemic Adverse Events: 9 μg ID vs 15 μg IM **eFigure 29.** Systemic Adverse Events: 15 μg ID vs 15 μg IM **eFigure 30.** Funnel Plot of Seroconversion: 9 μg ID vs 15 μg IM **eFigure 31.** Funnel Plot of Seroprotection: 9 μg vs 15 μg **eFigure 32.** Funnel Plot of GMT: 9 μg ID vs 15 μg IM **eFigure 33.** Funnel Plot of Seroconversion: 15 μg ID vs 15 μg IM **eFigure 34.** Funnel Plot of Seroprotection: 15 μg ID vs 15 μg IM **eFigure 35.** Funnel Plot of GMT: 15 μg ID vs 15 μg IM
