# Supplementary material for: Immunogenicity and Safety of Reduced-Dose Intradermal vs Intramuscular Influenza Vaccines: A Systematic Review and Meta-analysis
Source: JAMA Netw Open. 2021 Feb 9;4(2):e2035693. doi: 10.1001/jamanetworkopen.2020.35693 (PMC7873776; doi:10.1001/jamanetworkopen.2020.35693)
Supplement: Supplement. — eAppendix. Search Strategy eFigure 1. Quality Assessment of Randomized Clinical Trials eFigure 2. Seroconversion: 3 μg ID vs 15 μg IM eFigure 3. Seroconversion: 6 μg ID vs 15 μg IM eFigure 4. Seroconversion: 7.5 μg ID vs 15 μg IM eFigure 5. Seroconversion: 9 μg ID vs 15 μg IM in All Population eFigure 6. Seroconversion: in Older Adults 9 μg ID vs 15 μg IM eFigure 7. Seroconversion: 15 μg ID vs 15 μg IM eFigure 8. Seroconversion: in Older Adults 15 μg ID vs 15 μg IM eFigure 9. Seroprotection: 3 μg ID vs 15 μg IM eFigure 10. Seroprotection: 6 μg ID vs 15 μg IM eFigure 11. Seroprotection: 7.5 μg ID vs 15 μg IM eFigure 12. Seroprotection: 9 μg ID vs 15 μg IM eFigure 13. Seroprotection Older Adults: 9 μg ID vs 15 μg IM eFigure 14. Seroprotection: 15 μg ID vs 15 μg IM eFigure 15. Seroprotection in Older Adults: 15 μg ID vs 15 μg IM eFigure 16. GMT: 3 μg ID vs 15 μg IM eFigure 17. GMT: 6 μg ID vs 15 μg IM eFigure 18. GMT: 9 μg ID vs 15 μg IM eFigure 19. GMT: in Older Adults 9 μg ID vs 15 μg IM eFigure 20. GMT: 15 μg ID vs 15 μg IM eFigure 21. GMT in Older Adults: 15 μg ID vs 15 μg IM eFigure 22. Local Adverse Events: 3 μg ID vs 15 μg IM eFigure 23. Local Adverse Events: 6 μg ID vs 15 μg IM eFigure 24. Local Adverse Events: 9 μg ID vs 15 μg IM eFigure 25. Local Adverse Events: 15 μg ID vs 15 μg IM eFigure 26. Systemic Adverse Events: 3 μg ID vs 15 μg IM eFigure 27. Systemic Adverse Events: 6 μg ID vs 15 μg IM eFigure 28. Systemic Adverse Events: 9 μg ID vs 15 μg IM eFigure 29. Systemic Adverse Events: 15 μg ID vs 15 μg IM eFigure 30. Funnel Plot of Seroconversion: 9 μg ID vs 15 μg IM eFigure 31. Funnel Plot of Seroprotection: 9 μg vs 15 μg eFigure 32. Funnel Plot of GMT: 9 μg ID vs 15 μg IM eFigure 33. Funnel Plot of Seroconversion: 15 μg ID vs 15 μg IM eFigure 34. Funnel Plot of Seroprotection: 15 μg ID vs 15 μg IM eFigure 35. Funnel Plot of GMT: 15 μg ID vs 15 μg IM [file jamanetwopen-e2035693-s001.pdf]

Supplemental Online Content

Egunsola O, Clement F, Taplin J, et al. Immunogenicity and safety of reduced-dose intradermal vs intramuscular influenza vaccines: a systematic review and meta-analysis. *JAMA Netw Open.* 2021;4(2):e2035693. doi:10.1001/jamanetworkopen.2020.35693

eAppendix. Search Strategy
eFigure 1. Quality Assessment of Randomized Clinical Trials
eFigure 2. Seroconversion: 3 µg ID vs 15 µg IM
eFigure 3. Seroconversion: 6 µg ID vs 15 µg IM
eFigure 4. Seroconversion: 7.5 µg ID vs 15 µg IM
eFigure 5. Seroconversion: 9 µg ID vs 15 µg IM in All Population
eFigure 6. Seroconversion: in Older Adults 9 µg ID vs 15 µg IM
eFigure 7. Seroconversion: 15 µg ID vs 15 µg IM
eFigure 8. Seroconversion: in Older Adults 15 µg ID vs 15 µg IM
eFigure 9. Seroprotection: 3 µg ID vs 15 µg IM
eFigure 10. Seroprotection: 6 µg ID vs 15 µg IM
eFigure 11. Seroprotection: 7.5 µg ID vs 15 µg IM
eFigure 12. Seroprotection: 9 µg ID vs 15 µg IM
eFigure 13. Seroprotection Older Adults: 9 µg ID vs 15 µg IM
eFigure 14. Seroprotection: 15 µg ID vs 15 µg IM
eFigure 15. Seroprotection in Older Adults: 15 µg ID vs 15 µg IM
eFigure 16. GMT: 3 µg ID vs 15 µg IM
eFigure 17. GMT: 6 µg ID vs 15 µg IM
eFigure 18. GMT: 9 µg ID vs 15 µg IM
eFigure 19. GMT: in Older Adults 9 µg ID vs 15 µg IM
eFigure 20. GMT: 15 µg ID vs 15 µg IM
eFigure 21. GMT in Older Adults: 15 µg ID vs 15 µg IM
eFigure 22. Local Adverse Events: 3 µg ID vs 15 µg IM
eFigure 23. Local Adverse Events: 6 µg ID vs 15 µg IM
eFigure 24. Local Adverse Events: 9 µg ID vs 15 µg IM
eFigure 25. Local Adverse Events: 15 µg ID vs 15 µg IM
eFigure 26. Systemic Adverse Events: 3 µg ID vs 15 µg IM
eFigure 27. Systemic Adverse Events: 6 µg ID vs 15 µg IM
eFigure 28. Systemic Adverse Events: 9 µg ID vs 15 µg IM
eFigure 29. Systemic Adverse Events: 15 µg ID vs 15 µg IM
eFigure 30. Funnel Plot of Seroconversion: 9 µg ID vs 15 µg IM
eFigure 31. Funnel Plot of Seroprotection: 9 µg vs 15 µg
eFigure 32. Funnel Plot of GMT: 9 µg ID vs 15 µg IM
eFigure 33. Funnel Plot of Seroconversion: 15 µg ID vs 15 µg IM
eFigure 34. Funnel Plot of Seroprotection: 15 µg ID vs 15 µg IM
eFigure 35. Funnel Plot of GMT: 15 µg ID vs 15 µg IM

This supplemental material has been provided by the authors to give readers additional information about their work.

© 2021 Egunsola O et al. *JAMA Network Open.*

eAppendix. Search Strategy

MEDLINE

1. Influenza, Human/

2. exp influenzavirus a/ or exp influenzavirus b/ or influenzavirus c/

3. Influenza Vaccines/

4. (influenza* or flu).tw,kf.

5. 1 or 2 or 3 or 4

6. Injections, Intradermal/

7. (intradermal or intra-dermal).tw,kf.

8. ID injection*.tw,kf.

9. IDflu.tw,kf.

10. (inject* adj5 (dermal or dermis)).tw,kf.

11. mantoux.tw,kf.

12. dose sparing.tw,kf.

13. 6 or 7 or 8 or 9 or 10 or 11 or 12

14. 5 and 13

15. animals/ not humans/

16. 14 not 15

17. limit 16 to (case reports or editorial or letter)

18. 16 not 17

19. limit 18 to yr="2010 -Current"

EMBASE

1. influenza/ or exp influenza a/ or influenza b/ or influenza c/ or seasonal influenza/

2. influenza virus/ or exp influenzavirus a/ or exp influenzavirus b/ or exp influenzavirus c/

© 2021 Egunsola O et al. *JAMA Network Open.*

3. influenza vaccine/

4. (influenza* or flu).tw,kw.

5. 1 or 2 or 3 or 4

6. intradermal drug administration/

7. (intradermal or intra-dermal).tw,kw.

8. ID injection*.tw,kw.

9. IDflu.tw,kw.

10. (inject* adj5 (dermal or dermis)).tw,kw.

11. mantoux.tw,kw.

12. dose sparing.tw,kw.

13. 6 or 7 or 8 or 9 or 10 or 11 or 12

14. 5 and 13

15. exp influenza vaccine/dl [Intradermal Drug Administration]

16. 14 or 15

17. animals/ not human/

18. 16 not 17

19. limit 18 to (conference abstract or editorial or letter)

20. 18 not 19

21. limit 20 to yr="2010 -Current"

Cochrane Central Register of Controlled Trials

1. Influenza, Human/

2. exp influenzavirus a/ or exp influenzavirus b/ or influenzavirus c/

3. Influenza Vaccines/

4. (influenza* or flu).tw.

© 2021 Egunsola O et al. *JAMA Network Open.*

5. 1 or 2 or 3 or 4

6. Injections, Intradermal/

7. (intradermal or intra-dermal).tw.

8. ID injection*.tw.

9. IDflu.tw.

10. (inject* adj5 (dermal or dermis)).tw.

11. mantoux.tw.

12. dose sparing.tw.

13. 6 or 7 or 8 or 9 or 10 or 11 or 12

14. 5 and 13

15. animals/ not humans/

16. 14 not 15

© 2021 Egunsola O et al. *JAMA Network Open.*

eFigure 1. Quality Assessment of Randomized Clinical Trials

Author (Year)	Domain 1	Domain 2	Domain 3	Domain 4	Domain 5	Overall Bias
Ansaldi et al. (2013)[18]	Some Concern	Some Concern	Low Concern	Low Concern	Some Concern	Some Concern
Ansaldi et al. (2012)[17]	Some Concern	Some Concern	Low Concern	Low Concern	Some Concern	Some Concern
Arnou et al. (2010)[19]	Low Concern	Some Concern	Low Concern	Low Concern	Some Concern	Some Concern
Boonak et al. (2017)[20]	Some Concern	Some Concern	Low Concern	Low Concern	Some Concern	Some Concern
Camiloni et al. (2014)[21]	Some Concern	Some Concern	Low Concern	Low Concern	Some Concern	Some Concern
Carter et al. (2019)[22]	Low Concern	Low Concern	Low Concern	Low Concern	Some Concern	Some Concern
Chan et al. (2014)[23]	Low Concern	Some Concern	Low Concern	Low Concern	Some Concern	Some Concern
Chi et al. (2010)[24]	Some Concern	Some Concern	Low Concern	Low Concern	Some Concern	Some Concern
Chuaychoo et al.	Some Concern	Some Concern	Low	Low	Some Concern	Some Concern

© 2021 Egunsola O et al. *JAMA Network Open.*

Author (Year)	Domain 1	Domain 2	Domain 3	Domain 4	Domain 5	Overall Bias
(2019)[25]			Concern	Concern		
Chuaychoo et al. (2016)[26]	Some Concern	Some Concern	Low Concern	Low Concern	Some Concern	Some Concern
Chuaychoo et al. (2010)[27]	High Concern	Some Concern	Low Concern	Low Concern	Some Concern	High Concern
Della Cioppa et al. (2014)[28]	Some Concern	Some Concern	Low Concern	Low Concern	Some Concern	Some Concern
Esposito et al. (2011)[29]	Low Concern	Some Concern	Low Concern	Low Concern	Some Concern	Some Concern
Frenck et al. (2011)[30]	Some Concern	Some Concern	Low Concern	Low Concern	Some Concern	Some Concern
Garg et al. (2016)[31]	Low Concern	Some Concern	Low Concern	Low Concern	Some Concern	Some Concern
Gorse et al. (2013)[32]	Some Concern	Some Concern	Low Concern	Low Concern	Some Concern	Some Concern
Han et al. (2013)[33]	High Concern	Some Concern	Low Concern	High Concern	Some Concern	High Concern
Hung et al. (2016)[34]	Low Concern	Low Concern	Low Concern	Low Concern	Some Concern	Some Concern

© 2021 Egunsola O et al. *JAMA Network Open.*

Author (Year)	Domain 1	Domain 2	Domain 3	Domain 4	Domain 5	Overall Bias
Hung et al. (2014)[36]	Some Concern	Some Concern	Low Concern	Low Concern	Some Concern	Some Concern
Hung et al. (2012)[35]	Some Concern	Some Concern	Low Concern	Low Concern	Some Concern	Some Concern
Leung et al. (2017)[37]	Some Concern	Some Concern	Low Concern	Low Concern	Some Concern	Some Concern
Levin et al. (2016)[45]	Some Concern	Some Concern	Low Concern	Low Concern	Some Concern	Some Concern
Levin et al. (2014)[44]	Some Concern	Some Concern	Low Concern	Low Concern	Some Concern	Some Concern
Nougaredet al. (2014)[38]	Some Concern	Some Concern	Low Concern	Low Concern	Some Concern	Some Concern
Patel et al. (2010)[39]	Some Concern	Some Concern	Low Concern	Low Concern	Some Concern	Some Concern
Seo et al. (2014)[40]	Some Concern	Some Concern	Low Concern	Low Concern	Some Concern	Some Concern
Song et al. (2013)[41]	Some Concern	Some Concern	Low Concern	Low Concern	Some Concern	Some Concern
Tsang et al.	Some Concern	Some Concern	Low	Low	Some Concern	Some Concern

© 2021 Egunsola O et al. *JAMA Network Open.*

Author (Year)	Domain 1	Domain 2	Domain 3	Domain 4	Domain 5	Overall Bias
(2014)[42]			Concern	Concern		
Van Damme et al. (2010)[43]	Some Concern	Some Concern	Low Concern	Low Concern	Some Concern	Some Concern

© 2021 Egunsola O et al. *JAMA Network Open.*

eFigure 2. Seroconversion: 3 µg ID vs 15 µg IM

Author	Intradermal Events	Intradermal Sample Size	Intramuscular Events	Intramuscular Sample Size	RR	95%-CI
**Strain = A/H1N1**						
Hung 2012	15	63	4	66	3.93	[1.38; 11.20]
Song 2013	13	30	15	32	0.92	[0.53; 1.60]
**Random effect model**		**93**		**98**	**1.77**	**[0.43; 7.27]**
Heterogeneity: I^2 = 83%, p = 0.02						
**Strain = A/H3N2**						
Hung 2012	41	63	27	66	1.59	[1.13; 2.24]
Song 2013	13	30	18	32	0.77	[0.46; 1.28]
**Random effect model**		**93**		**98**	**1.14**	**[0.56; 2.31]**
Heterogeneity: I^2 = 81%, p = 0.02						
**Strain = B Strain**						
Hung 2012	27	63	19	66	1.49	[0.93; 2.39]
Song 2013	12	30	15	32	0.85	[0.48; 1.51]
**Random effect model**		**93**		**98**	**1.15**	**[0.67; 1.99]**
Heterogeneity: I^2 = 53%, p = 0.14						

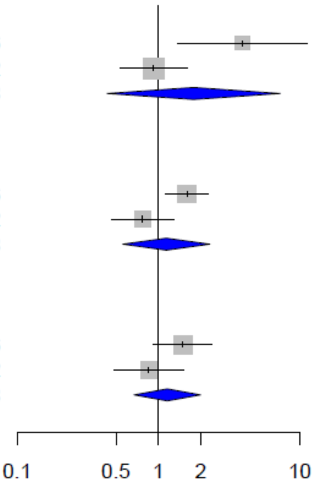

eFigure 3. Seroconversion: 6 µg ID vs 15 µg IM

Author	Intradermal Events	Intradermal Sample Size	Intramuscular Events	Intramuscular Sample Size	RR	95%-CI
**Strain = A/H1N1**						
Levin 2016	30	61	27	63	1.15	[0.78; 1.68]
Levin 2016*	30	61	32	63	0.97	[0.68; 1.38]
Song 2013	12	30	15	32	0.85	[0.48; 1.51]
**Random effect model**		**152**		**158**	**1.01**	**[0.80; 1.28]**
Heterogeneity: I^2 = 0% [0%; 74%], p = 0.67						
**Strain = A/H3N2**						
Levin 2016	30	61	24	63	1.29	[0.86; 1.94]
Levin 2016*	30	61	44	63	0.70	[0.52; 0.95]
Song 2013	15	30	18	32	0.89	[0.56; 1.42]
**Random effect model**		**152**		**158**	**0.92**	**[0.63; 1.33]**
Heterogeneity: I^2 = 64% [0%; 90%], p = 0.06						
**Strain = B Strain**						
Levin 2016	22	61	12	63	1.89	[1.03; 3.48]
Levin 2016*	22	61	20	63	1.14	[0.69; 1.86]
Song 2013	12	30	15	32	0.85	[0.48; 1.51]
**Random effect model**		**152**		**158**	**1.21**	**[0.79; 1.85]**
Heterogeneity: I^2 = 44% [0%; 83%], p = 0.17						

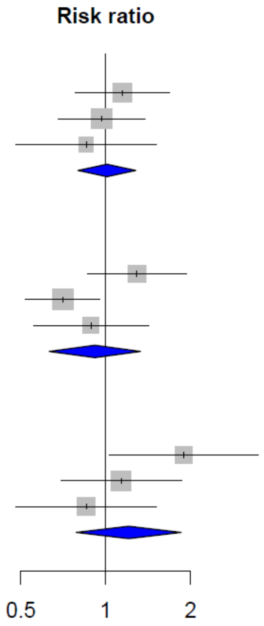

© 2021 Egunsola O et al. *JAMA Network Open.*

eFigure 4. Seroconversion: 7.5 µg ID vs 15 µg IM

Author	Intradermal Events	Intradermal Sample Size	Intramuscular Events	Intramuscular Sample Size	RR	95%-CI
**Strain = A/H1N1**						
Levin 2016	30	61	27	63	1.15	[0.78; 1.68]
Levin 2016*	30	61	32	63	0.97	[0.68; 1.38]
Song 2013	12	30	15	32	0.85	[0.48; 1.51]
**Random effect model**		**152**		**158**	**1.01**	**[0.80; 1.28]**
Heterogeneity: $I^2 = 0\%$ [0%; 74%], $p = 0.67$						
**Strain = A/H3N2**						
Levin 2016	30	61	24	63	1.29	[0.86; 1.94]
Levin 2016*	30	61	44	63	0.70	[0.52; 0.95]
Song 2013	15	30	18	32	0.89	[0.56; 1.42]
**Random effect model**		**152**		**158**	**0.92**	**[0.63; 1.33]**
Heterogeneity: $I^2 = 64\%$ [0%; 90%], $p = 0.06$						
**Strain = B Strain**						
Levin 2016	22	61	12	63	1.89	[1.03; 3.48]
Levin 2016*	22	61	20	63	1.14	[0.69; 1.86]
Song 2013	12	30	15	32	0.85	[0.48; 1.51]
**Random effect model**		**152**		**158**	**1.21**	**[0.79; 1.85]**
Heterogeneity: $I^2 = 44\%$ [0%; 83%], $p = 0.17$						

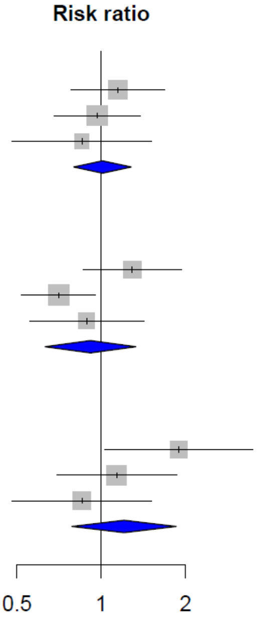

© 2021 Egunsola O et al. *JAMA Network Open.*

eFigure 5. Seroconversion: 9 µg ID vs 15 µg IM in All Population
† Soluvia Microneedle

Author	Intradermal Events	Intradermal Sample Size	Intramuscular Events	Intramuscular Sample Size	RR	95%-CI
**Strain = A/H1N1**						
Arnou 2010	722	1255	237	421	1.02	[0.93; 1.13]
Carter 2019	22	49	26	56	0.97	[0.64; 1.47]
Chuaychoo 2016	38	75	49	74	0.77	[0.58; 1.01]
Chuaychoo 2019	34	41	24	39	1.35	[1.01; 1.79]
Esposito 2011	36	38	33	37	1.06	[0.93; 1.22]
Gorse 2013	1577	2581	776	1287	1.01	[0.96; 1.07]
Han 2013	49	60	55	60	0.89	[0.77; 1.03]
Hung 2012	17	68	4	66	4.12	[1.47; 11.61]
Hung 2012†	11	65	4	66	2.79	[0.94; 8.32]
Nougarede 2014	25	38	26	42	1.06	[0.76; 1.48]
**Random effect model**		**4270**		**2148**	**1.02**	**[0.93; 1.12]**
Heterogeneity: $I^2 = 59\%$ [18%; 80%], $p < 0.01$						
**Strain = A/H3N2**						
Arnou 2010	835	1255	292	421	0.96	[0.89; 1.03]
Carter 2019	20	49	34	56	0.67	[0.45; 1.00]
Chuaychoo 2016	45	75	41	74	1.08	[0.82; 1.43]
Chuaychoo 2019	27	41	27	39	0.95	[0.70; 1.29]
Esposito 2011	37	38	36	37	1.00	[0.93; 1.08]
Gorse 2013	1938	2581	960	1287	1.01	[0.97; 1.05]
Han 2013	52	60	52	60	1.00	[0.87; 1.15]
Hung 2012	41	68	27	66	1.47	[1.04; 2.09]
Hung 2012†	38	65	27	66	1.43	[1.00; 2.04]
Nougarede 2014	25	38	25	42	1.11	[0.79; 1.55]
**Random effect model**		**4270**		**2148**	**1.01**	**[0.95; 1.06]**
Heterogeneity: $I^2 = 38\%$ [0%; 70%], $p = 0.10$						
**Strain = B Strain**						
Arnou 2010	712	1255	256	421	0.93	[0.85; 1.02]
Carter 2019	15	49	31	56	0.55	[0.34; 0.90]
Chuaychoo 2016	20	75	18	74	1.10	[0.63; 1.90]
Chuaychoo 2019	3	41	5	39	0.57	[0.15; 2.23]
Esposito 2011	22	38	12	37	1.79	[1.04; 3.06]
Gorse 2013	1189	2581	696	1287	0.85	[0.80; 0.91]
Han 2013	31	60	36	60	0.86	[0.63; 1.19]
Hung 2012	30	68	19	66	1.53	[0.96; 2.44]
Hung 2012†	22	65	19	66	1.18	[0.71; 1.96]
Nougarede 2014	24	38	26	42	1.02	[0.73; 1.43]
**Random effect model**		**4270**		**2148**	**0.95**	**[0.84; 1.08]**
Heterogeneity: $I^2 = 57\%$ [13%; 79%], $p = 0.01$						

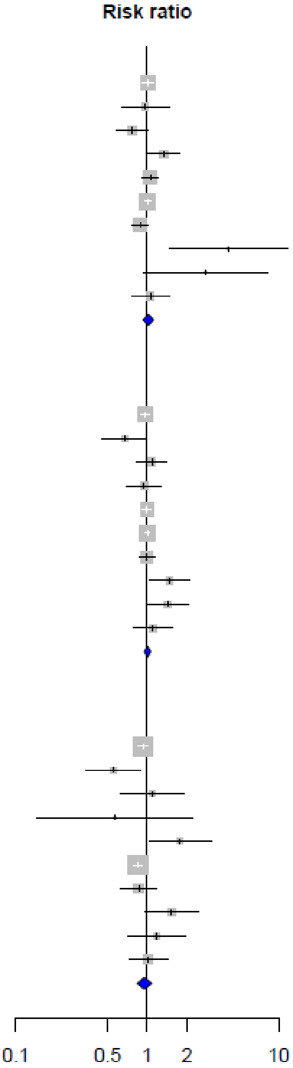

© 2021 Egunsola O et al. *JAMA Network Open.*

eFigure 6. Seroconversion: in Older Adults 9 µg ID vs 15 µg IM

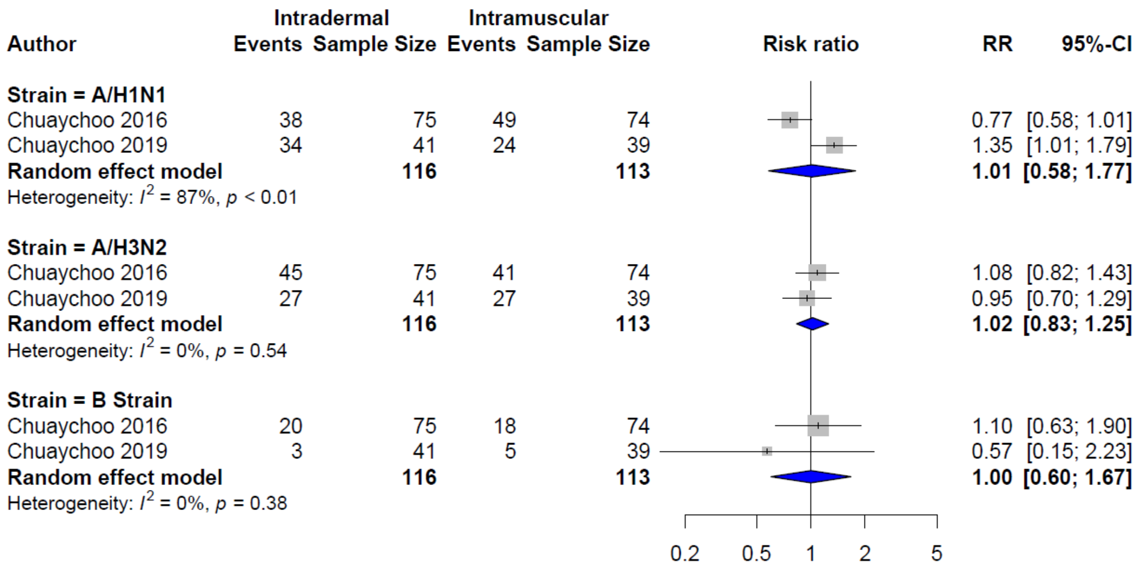

© 2021 Egunsola O et al. *JAMA Network Open.*

eFigure 7. Seroconversion: 15 µg ID vs 15 µg IM
(*Inflexa vs Inflexa, ** Intanza vs Inflexa,†Inflexa vs Fluad, ‡Intanza vs Fluad, ɫ Adjuvanted)

Author	Intradermal Events	Intradermal Sample Size	Intramuscular Events	Intramuscular Sample Size	RR	95%-CI
**Strain = A/H1N1**						
Ansaldi 2013	12	24	10	23	1.15	[0.62; 2.13]
Camiloni 2014	17	40	20	40	0.85	[0.53; 1.37]
Chan 2014	30	50	18	50	1.67	[1.08; 2.57]
Esposito 2011	36	37	33	37	1.09	[0.96; 1.24]
Garg 2015	21	40	24	40	0.88	[0.59; 1.29]
Han 2013	50	60	47	60	1.06	[0.89; 1.27]
Hung 2014	18	31	11	30	1.58	[0.91; 2.77]
Hung 2016	28	40	22	40	1.27	[0.90; 1.80]
Levin 2016*	40	61	27	63	1.53	[1.09; 2.15]
Levin 2016**	32	60	27	63	1.24	[0.86; 1.80]
Levin 2016†	40	61	32	63	1.29	[0.95; 1.75]
Levin 2016‡	32	60	32	63	1.05	[0.75; 1.47]
Seo 2014	47	111	44	113	1.09	[0.79; 1.49]
Seo 2014ɫ	47	111	60	111	0.78	[0.59; 1.03]
Tsang 2014	453	636	193	317	1.17	[1.06; 1.29]
Van Damme 2010	272	395	287	395	0.95	[0.87; 1.04]
**Random effect model**		**1817**		**1508**	**1.10**	**[1.01; 1.20]**
Heterogeneity: I^2 = 50% [12%; 72%], p = 0.01						
**Strain = A/H3N2**						
Ansaldi 2012	15	25	15	25	1.00	[0.64; 1.57]
Ansaldi 2013	12	24	8	23	1.44	[0.72; 2.86]
Camiloni 2014	24	40	19	40	1.26	[0.84; 1.91]
Chan 2014	15	50	8	50	1.88	[0.87; 4.02]
Esposito 2011	37	37	36	37	1.03	[0.97; 1.08]
Garg 2015	22	40	23	40	0.96	[0.65; 1.41]
Han 2013	50	60	47	60	1.06	[0.89; 1.27]
Hung 2014	11	31	5	30	2.13	[0.84; 5.40]
Hung 2016	7	40	7	40	1.00	[0.39; 2.59]
Levin 2016*	37	61	24	63	1.59	[1.10; 2.31]
Levin 2016**	35	60	24	63	1.53	[1.05; 2.24]
Levin 2016†	37	61	44	63	0.87	[0.67; 1.13]
Levin 2016‡	35	60	44	63	0.84	[0.64; 1.09]
Seo 2014	48	111	30	113	1.63	[1.12; 2.37]
Seo 2014ɫ	48	111	50	111	0.96	[0.71; 1.29]
Tsang 2014	263	636	120	317	1.09	[0.92; 1.29]
Van Damme 2010	186	395	198	395	0.94	[0.81; 1.08]
**Random effect model**		**1842**		**1533**	**1.07**	**[0.99; 1.17]**
Heterogeneity: I^2 = 43% [0%; 68%], p = 0.03						
**Strain = B Strain**						
Ansaldi 2013	11	24	0	23	22.06	[1.38; 353.58]
Camiloni 2014	16	40	4	40	4.00	[1.47; 10.92]
Chan 2014	10	50	3	50	3.33	[0.98; 11.40]
Esposito 2011	23	37	12	37	1.92	[1.13; 3.25]
Garg 2015	19	40	24	40	0.79	[0.52; 1.20]
Han 2013	38	60	39	60	0.97	[0.75; 1.27]
Hung 2014	3	31	5	30	0.58	[0.15; 2.22]
Hung 2016	27	40	20	40	1.35	[0.93; 1.97]
Levin 2016*	21	61	12	63	1.81	[0.98; 3.35]
Levin 2016**	28	60	12	63	2.45	[1.38; 4.36]
Levin 2016†	21	61	20	63	1.08	[0.66; 1.79]
Levin 2016‡	28	60	20	63	1.47	[0.94; 2.31]
Seo 2014	8	111	2	113	4.07	[0.88; 18.75]
Seo 2014ɫ	8	111	8	111	1.00	[0.39; 2.57]
Tsang 2014	92	636	31	317	1.48	[1.01; 2.17]
Van Damme 2010	91	395	89	395	1.02	[0.79; 1.32]
**Random effect model**		**1817**		**1508**	**1.40**	**[1.13; 1.73]**
Heterogeneity: I^2 = 59% [29%; 76%], p < 0.01						

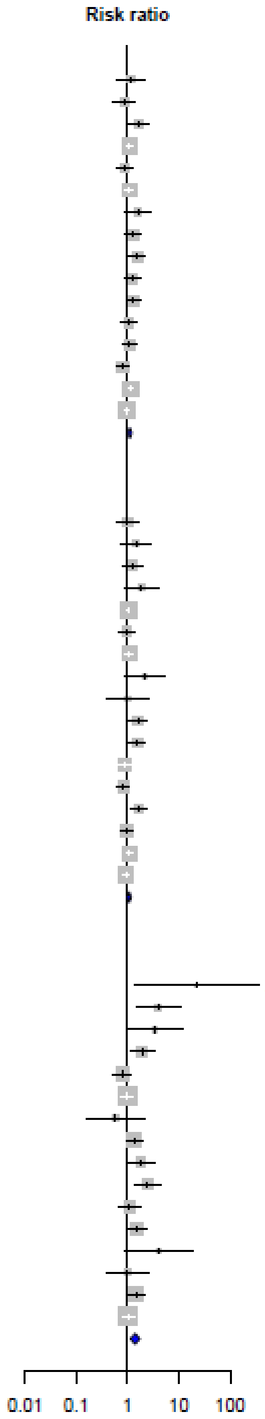

© 2021 Egunsola O et al. *JAMA Network Open.*

eFigure 8. Seroconversion: in Older Adults 15 μg ID vs 15 μg IM
(*Inflexa vs Inflexa, ** Intanza vs Inflexa,†Inflexa vs Fluad, ‡Intanza vs Fluad, ɫ Adjuvanted)

Author	Intradermal Events	Intradermal Sample Size	Intramuscular Events	Intramuscular Sample Size	RR	95%-CI
**Strain = A/H1N1**						
Ansaldi 2013	12	24	10	23	1.15	[0.62; 2.13]
Boonnak 2017	58	111	44	110	1.31	[0.98; 1.75]
Camiloni 2014	17	40	20	40	0.85	[0.53; 1.37]
Chan 2014	30	50	18	50	1.67	[1.08; 2.57]
Han 2013†	50	60	47	60	1.06	[0.89; 1.27]
Levin 2016*	40	61	27	63	1.53	[1.09; 2.15]
Levin 2016**	32	60	27	63	1.24	[0.86; 1.80]
Levin 2016†	40	61	32	63	1.29	[0.95; 1.75]
Levin 2016‡	32	60	32	63	1.05	[0.75; 1.47]
Seo 2014	47	111	44	113	1.09	[0.79; 1.49]
Seo 2014ɫ	47	111	60	111	0.78	[0.59; 1.03]
Tsang 2014	453	636	193	317	1.17	[1.06; 1.29]
Van Damme 2010	272	395	287	395	0.95	[0.87; 1.04]
**Random effect model**		**1780**		**1471**	**1.11**	**[1.00; 1.24]**
Heterogeneity: I^2 = 57% [21%; 77%], p < 0.01						
**Strain = A/H3N2**						
Ansaldi 2012	15	25	15	25	1.00	[0.64; 1.57]
Ansaldi 2013	12	24	8	23	1.44	[0.72; 2.86]
Boonnak 2017	68	111	54	110	1.25	[0.98; 1.59]
Camiloni 2014	24	40	19	40	1.26	[0.84; 1.91]
Chan 2014	15	50	8	50	1.88	[0.87; 4.02]
Han 2013†	50	60	47	60	1.06	[0.89; 1.27]
Levin 2016*	37	61	24	63	1.59	[1.10; 2.31]
Levin 2016**	35	60	24	63	1.53	[1.05; 2.24]
Levin 2016†	37	61	44	63	0.87	[0.67; 1.13]
Levin 2016‡	35	60	44	63	0.84	[0.64; 1.09]
Seo 2014	48	111	30	113	1.63	[1.12; 2.37]
Seo 2014ɫ	48	111	50	111	0.96	[0.71; 1.29]
Tsang 2014	263	636	120	317	1.09	[0.92; 1.29]
Van Damme 2010	186	395	198	395	0.94	[0.81; 1.08]
**Random effect model**		**1805**		**1496**	**1.12**	**[1.00; 1.25]**
Heterogeneity: I^2 = 52% [12%; 74%], p = 0.01						
**Strain = B Strain**						
Ansaldi 2013	11	24	0	23	22.06	[1.38; 353.58]
Boonnak 2017	68	111	62	110	1.09	[0.87; 1.36]
Camiloni 2014	16	40	4	40	4.00	[1.47; 10.92]
Chan 2014	10	50	3	50	3.33	[0.98; 11.40]
Han 2013†	38	60	39	60	0.97	[0.75; 1.27]
Levin 2016*	21	61	12	63	1.81	[0.98; 3.35]
Levin 2016**	28	60	12	63	2.45	[1.38; 4.36]
Levin 2016†	21	61	20	63	1.08	[0.66; 1.79]
Levin 2016‡	28	60	20	63	1.47	[0.94; 2.31]
Seo 2014	8	111	2	113	4.07	[0.88; 18.75]
Seo 2014ɫ	8	111	8	111	1.00	[0.39; 2.57]
Tsang 2014	92	636	31	317	1.48	[1.01; 2.17]
Van Damme 2010	91	395	89	395	1.02	[0.79; 1.32]
**Random effect model**		**1780**		**1471**	**1.41**	**[1.13; 1.75]**
Heterogeneity: I^2 = 59% [24%; 78%], p < 0.01						

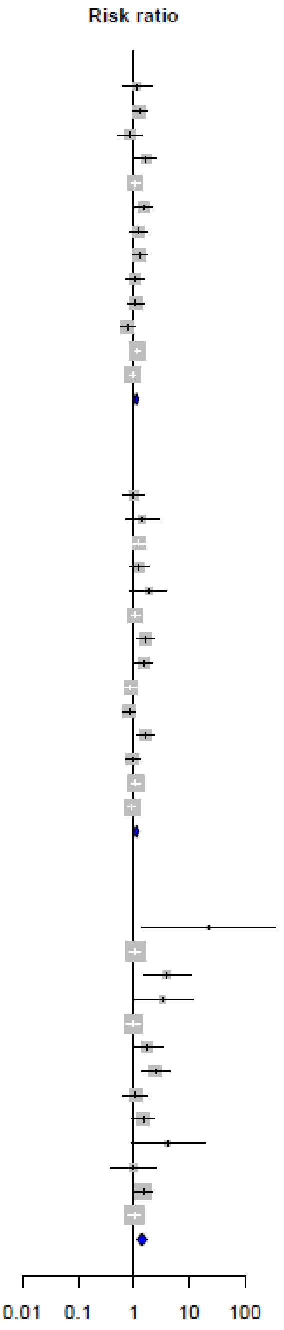

© 2021 Egunsola O et al. *JAMA Network Open.*

eFigure 9. Seroprotection: 3 μg ID vs 15 μg IM

Author	Intradermal Events	Intradermal Sample Size	Intramuscular Events	Intramuscular Sample Size	RR	95%-CI
**Strain = A/H1N1**						
Frenck 2011	291	395	336	394	0.86	[0.80; 0.93]
Hung 2012	57	63	47	66	1.27	[1.07; 1.51]
Song 2013	24	30	28	32	0.91	[0.73; 1.14]
**Random effect model**		**488**		**492**	**1.00**	**[0.78; 1.28]**
Heterogeneity: I^2 = 88% [65%; 96%], p < 0.01						
**Strain = A/H3N2**						
Frenck 2011	387	395	393	394	0.98	[0.97; 1.00]
Hung 2012	57	63	61	66	0.98	[0.88; 1.09]
Song 2013	21	30	27	32	0.83	[0.63; 1.10]
**Random effect model**		**488**		**492**	**0.98**	**[0.97; 1.00]**
Heterogeneity: I^2 = 0% [0%; 85%], p = 0.49						
**Strain = B Strain**						
Frenck 2011	255	395	320	394	0.79	[0.73; 0.87]
Hung 2012	53	63	44	66	1.26	[1.03; 1.54]
Song 2013	18	30	23	32	0.83	[0.58; 1.20]
**Random effect model**		**488**		**492**	**0.94**	**[0.68; 1.32]**
Heterogeneity: I^2 = 88% [67%; 96%], p < 0.01						

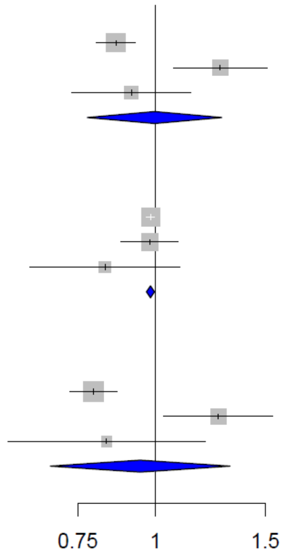

eFigure 10. Seroprotection: 6 μg ID vs 15 μg IM

Author	Intradermal Events	Intradermal Sample Size	Intramuscular Events	Intramuscular Sample Size	RR	95%-CI
**Strain = A/H1N1**						
Chuaychoo 2010	75	81	70	75	0.99	[0.91; 1.08]
Frenck 2011	300	392	336	394	0.90	[0.84; 0.96]
Levin 2014	49	55	52	54	0.93	[0.83; 1.03]
**Random effect model**		**528**		**523**	**0.93**	**[0.88; 0.99]**
Heterogeneity: I^2 = 37% [0%; 80%], p = 0.20						
**Strain = A/H3N2**						
Chuaychoo 2010	71	81	66	75	1.00	[0.89; 1.12]
Della Chioppa 2014	88	43	93	43		
Frenck 2011	391	392	393	394	1.00	[0.99; 1.01]
Levin 2014	54	55	51	54	1.04	[0.97; 1.12]
**Random effect model**		**571**		**566**	**1.00**	**[0.99; 1.01]**
Heterogeneity: I^2 = 0% [0%; 80%], p = 0.59						
**Strain = B Strain**						
Chuaychoo 2010	55	81	54	75	0.94	[0.77; 1.16]
Frenck 2011	294	392	320	394	0.92	[0.86; 0.99]
Levin 2014	40	55	46	54	0.85	[0.70; 1.04]
**Random effect model**		**528**		**523**	**0.92**	**[0.86; 0.98]**
Heterogeneity: I^2 = 0% [0%; 66%], p = 0.74						

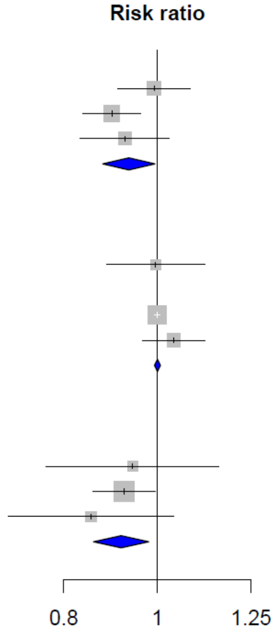

© 2021 Egunsola O et al. *JAMA Network Open.*

eFigure 11. Seroprotection: 7.5 μg ID vs 15 μg IM

Author	Intradermal Events	Intradermal Sample Size	Intramuscular Events	Intramuscular Sample Size	RR	95%-CI
**Strain = A/H1N1**						
Levin 2016	60	61	56	63	1.11	[1.01; 1.21]
Levin 2016*	60	61	59	63	1.05	[0.98; 1.13]
Song 2013	27	30	28	32	1.03	[0.86; 1.23]
**Random effect model**		**152**		**158**	**1.07**	**[1.01; 1.13]**
Heterogeneity: $I^2 = 0\%$ [0%; 78%], $p = 0.63$						
**Strain = A/H3N2**						
Levin 2016	60	61	59	63	1.05	[0.98; 1.13]
Levin 2016*	60	61	63	63	0.98	[0.95; 1.02]
Song 2013	27	30	27	32	1.07	[0.88; 1.29]
**Random effect model**		**152**		**158**	**1.01**	**[0.96; 1.06]**
Heterogeneity: $I^2 = 37\%$ [0%; 80%], $p = 0.21$						
**Strain = B Strain**						
Levin 2016	27	61	17	63	1.64	[1.00; 2.69]
Levin 2016*	27	61	24	63	1.16	[0.76; 1.77]
Song 2013	18	30	23	32	0.83	[0.58; 1.20]
**Random effect model**		**152**		**158**	**1.13**	**[0.78; 1.66]**
Heterogeneity: $I^2 = 58\%$ [0%; 88%], $p = 0.09$						

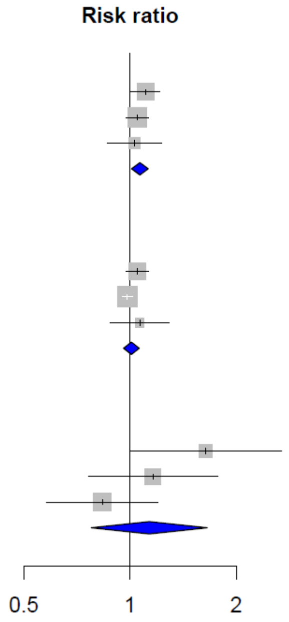

© 2021 Egunsola O et al. *JAMA Network Open.*

eFigure 12. Seroprotection: 9 µg ID vs 15 µg IM
(† Soluvia Microneedle, * Two Injections)

Author	Intradermal Events	Intradermal Sample Size	Intramuscular Events	Intramuscular Sample Size	RR	95%-CI
**Strain = A/H1N1**						
Arnou 2010	1094	1255	363	421	1.01	[0.97; 1.06]
Chi 2010	42	63	42	65	1.03	[0.80; 1.33]
Chi 2010*	43	65	42	65	1.02	[0.80; 1.31]
Chuaychoo 2016	48	75	58	74	0.82	[0.66; 1.01]
Chuaychoo 2019	39	41	36	39	1.03	[0.92; 1.16]
Esposito 2011	35	38	32	37	1.06	[0.91; 1.25]
Frenck 2011	316	390	336	394	0.95	[0.89; 1.01]
Gorse 2013	2350	2581	1176	1287	1.00	[0.98; 1.02]
Han 2013	55	60	59	60	0.93	[0.86; 1.01]
Hung 2012	59	68	47	66	1.22	[1.02; 1.46]
Hung 2012†	53	65	47	66	1.15	[0.94; 1.39]
Nougarede 2014	37	38	41	42	1.00	[0.93; 1.07]
**Random effect model**		**4739**		**2616**	**1.00**	**[0.97; 1.03]**
Heterogeneity: I^2 = 34% [0%; 67%], p = 0.12						
**Strain = A/H3N2**						
Arnou 2010	1173	1255	402	421	0.98	[0.95; 1.00]
Chi 2010	46	63	49	65	0.97	[0.79; 1.19]
Chi 2010*	48	65	49	65	0.98	[0.80; 1.20]
Chuaychoo 2016	61	75	59	74	1.02	[0.87; 1.19]
Chuaychoo 2019	38	41	33	39	1.10	[0.93; 1.28]
Esposito 2011	37	38	35	37	1.03	[0.94; 1.13]
Frenck 2011	388	390	393	394	1.00	[0.99; 1.01]
Gorse 2013	2335	2581	1174	1287	0.99	[0.97; 1.01]
Han 2013	59	60	59	60	1.00	[0.95; 1.05]
Hung 2012	62	68	61	66	0.99	[0.89; 1.09]
Hung 2012†	60	65	60	66	1.02	[0.92; 1.13]
Nougarede 2014	38	38	42	42	1.00	[0.95; 1.05]
**Random effect model**		**4739**		**2616**	**1.00**	**[0.99; 1.00]**
Heterogeneity: I^2 = 0% [0%; 0%], p = 0.96						
**Strain = B Strain**						
Arnou 2010	915	1255	315	421	0.97	[0.91; 1.04]
Chi 2010	10	63	17	65	0.61	[0.30; 1.22]
Chi 2010*	16	65	17	65	0.94	[0.52; 1.70]
Chuaychoo 2016	31	75	27	74	1.13	[0.76; 1.70]
Chuaychoo 2019	4	41	5	39	0.76	[0.22; 2.63]
Esposito 2011	21	38	12	37	1.70	[0.99; 2.94]
Frenck 2011	297	390	320	394	0.94	[0.87; 1.01]
Gorse 2013	2252	2581	1147	1287	0.98	[0.96; 1.00]
Han 2013	60	60	60	60	1.00	[0.97; 1.03]
Hung 2012	55	68	44	66	1.21	[0.99; 1.49]
Hung 2012†	53	65	44	66	1.22	[1.00; 1.50]
Nougarede 2014	29	38	40	42	0.80	[0.66; 0.97]
**Random effect model**		**4739**		**2616**	**0.99**	**[0.95; 1.03]**
Heterogeneity: I^2 = 50% [3%; 74%], p = 0.02						

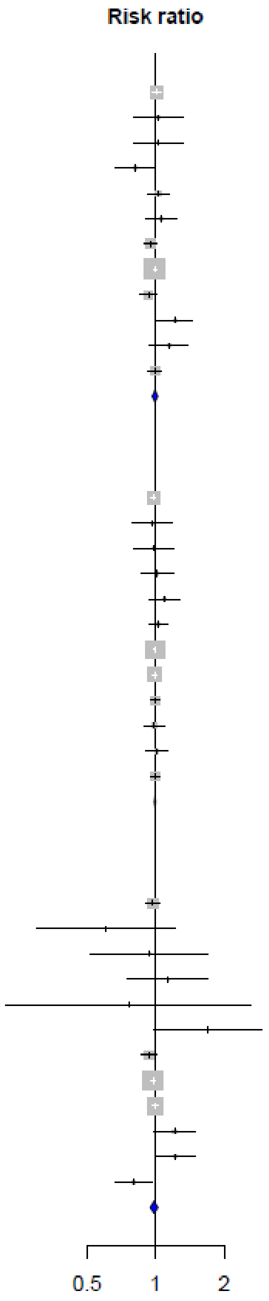

© 2021 Egunsola O et al. *JAMA Network Open.*

eFigure 13. Seroprotection Older Adults: 9 µg ID vs 15 µg IM
ǂ Two Injections

Author	Intradermal Events	Intradermal Sample Size	Intramuscular Events	Intramuscular Sample Size	RR	95%-CI
**Strain = A/H1N1**						
Chi 2010	42	63	42	65	1.03	[0.80; 1.33]
Chi 2010ǂ	43	65	42	65	1.02	[0.80; 1.31]
Chuaychoo 2016	48	75	58	74	0.82	[0.66; 1.01]
Chuaychoo 2019	39	41	36	39	1.03	[0.92; 1.16]
**Random effect model**		**244**		**243**	**0.98**	**[0.88; 1.09]**
Heterogeneity: I^2 = 24% [0%; 88%], p = 0.27						
**Strain = A/H3N2**						
Chi 2010	46	63	49	65	0.97	[0.79; 1.19]
Chi 2010ǂ	48	65	49	65	0.98	[0.80; 1.20]
Chuaychoo 2016	61	75	59	74	1.02	[0.87; 1.19]
Chuaychoo 2019	38	41	33	39	1.10	[0.93; 1.28]
**Random effect model**		**244**		**243**	**1.02**	**[0.94; 1.12]**
Heterogeneity: I^2 = 0% [0%; 60%], p = 0.76						
**Strain = B Strain**						
Chi 2010	10	63	17	65	0.61	[0.30; 1.22]
Chi 2010ǂ	16	65	17	65	0.94	[0.52; 1.70]
Chuaychoo 2016	31	75	27	74	1.13	[0.76; 1.70]
Chuaychoo 2019	4	41	5	39	0.76	[0.22; 2.63]
**Random effect model**		**244**		**243**	**0.95**	**[0.71; 1.27]**
Heterogeneity: I^2 = 0% [0%; 81%], p = 0.49						

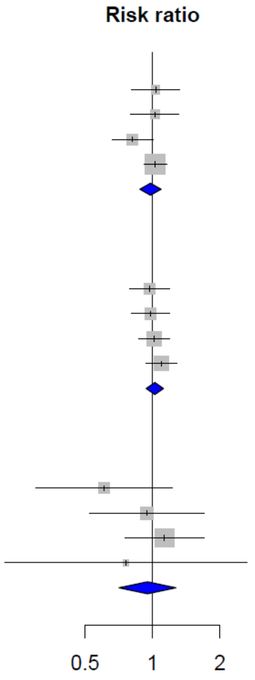

© 2021 Egunsola O et al. *JAMA Network Open.*

eFigure 14. Seroprotection: 15 μg ID vs 15 μg IM
(*Inflexa vs Inflexa, ** Intanza vs Inflexa,†Inflexa vs Fluad, ‡Intanza vs Fluad, ɫ Adjuvanted)

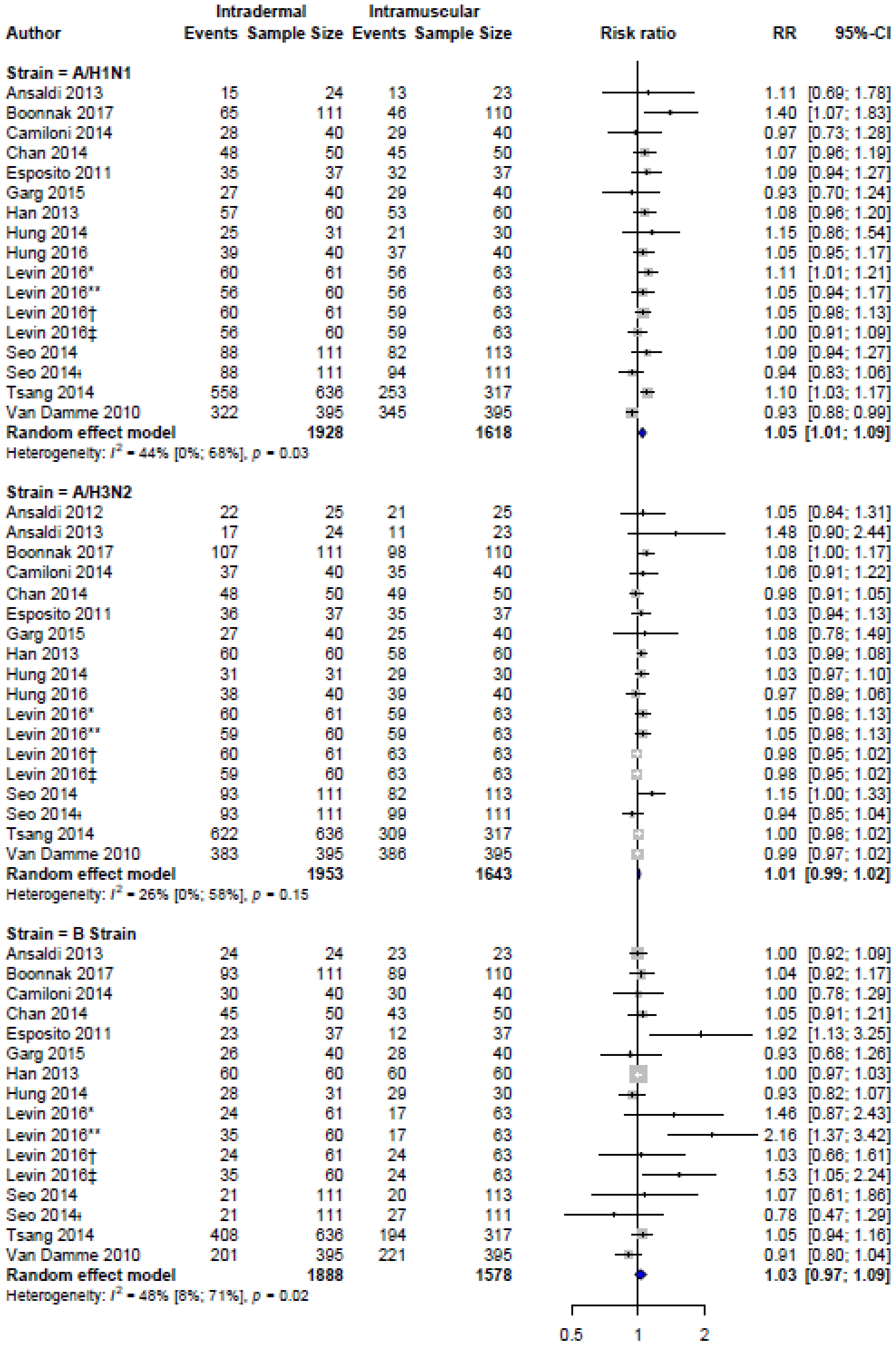

© 2021 Egunsola O et al. *JAMA Network Open.*

eFigure 15. Seroprotection in Older Adults: 15 µg ID vs 15 µg IM
(*Inflexa vs Inflexa, ** Intanza vs Inflexa,†Inflexa vs Fluad, ‡Intanza vs Fluad, ɫ Adjuvanted)

Author	Intradermal Events	Intradermal Sample Size	Intramuscular Events	Intramuscular Sample Size	RR	95%-CI
**Strain = A/H1N1**						
Ansaldi 2013	15	24	13	23	1.11	[0.69; 1.78]
Boonnak 2017	65	111	46	110	1.40	[1.07; 1.83]
Camiloni 2014	28	40	29	40	0.97	[0.73; 1.28]
Chan 2014	48	50	45	50	1.07	[0.96; 1.19]
Han 2013†	57	60	53	60	1.08	[0.96; 1.20]
Levin 2016*	60	61	56	63	1.11	[1.01; 1.21]
Levin 2016**	56	60	56	63	1.05	[0.94; 1.17]
Levin 2016†	60	61	59	63	1.05	[0.98; 1.13]
Levin 2016‡	56	60	59	63	1.00	[0.91; 1.09]
Seo 2014	88	111	82	113	1.09	[0.94; 1.27]
Seo 2014ɫ	88	111	94	111	0.94	[0.83; 1.06]
Tsang 2014	558	636	253	317	1.10	[1.03; 1.17]
Van Damme 2010	322	395	345	395	0.93	[0.88; 0.99]
**Random effect model**		**1780**		**1471**	**1.04**	**[1.00; 1.09]**
Heterogeneity: I^2 = 55% [16%; 76%], $p < 0.01$						
**Strain = A/H3N2**						
Ansaldi 2012	22	25	21	25	1.05	[0.84; 1.31]
Ansaldi 2013	17	24	11	23	1.48	[0.90; 2.44]
Boonnak 2017	107	111	98	110	1.08	[1.00; 1.17]
Camiloni 2014	37	40	35	40	1.06	[0.91; 1.22]
Chan 2014	48	50	49	50	0.98	[0.91; 1.05]
Han 2013†	60	60	58	60	1.03	[0.99; 1.08]
Levin 2016*	60	61	59	63	1.05	[0.98; 1.13]
Levin 2016**	59	60	59	63	1.05	[0.98; 1.13]
Levin 2016†	60	61	63	63	0.98	[0.95; 1.02]
Levin 2016‡	59	60	63	63	0.98	[0.95; 1.02]
Seo 2014	93	111	82	113	1.15	[1.00; 1.33]
Seo 2014ɫ	93	111	99	111	0.94	[0.85; 1.04]
Tsang 2014	622	636	309	317	1.00	[0.98; 1.02]
Van Damme 2010	383	395	386	395	0.99	[0.97; 1.02]
**Random effect model**		**1805**		**1496**	**1.01**	**[0.99; 1.03]**
Heterogeneity: I^2 = 39% [0%; 67%], p = 0.07						
**Strain = B Strain**						
Ansaldi 2013	24	24	23	23	1.00	[0.92; 1.09]
Boonnak 2017	93	111	89	110	1.04	[0.92; 1.17]
Camiloni 2014	30	40	30	40	1.00	[0.78; 1.29]
Chan 2014	45	50	43	50	1.05	[0.91; 1.21]
Han 2013†	60	60	60	60	1.00	[0.97; 1.03]
Levin 2016*	24	61	17	63	1.46	[0.87; 2.43]
Levin 2016**	35	60	17	63	2.16	[1.37; 3.42]
Levin 2016†	24	61	24	63	1.03	[0.66; 1.61]
Levin 2016‡	35	60	24	63	1.53	[1.05; 2.24]
Seo 2014	21	111	20	113	1.07	[0.61; 1.86]
Seo 2014ɫ	21	111	27	111	0.78	[0.47; 1.29]
Tsang 2014	408	636	194	317	1.05	[0.94; 1.16]
Van Damme 2010	201	395	221	395	0.91	[0.80; 1.04]
**Random effect model**		**1780**		**1471**	**1.03**	**[0.97; 1.09]**
Heterogeneity: I^2 = 45% [0%; 71%], p = 0.04						

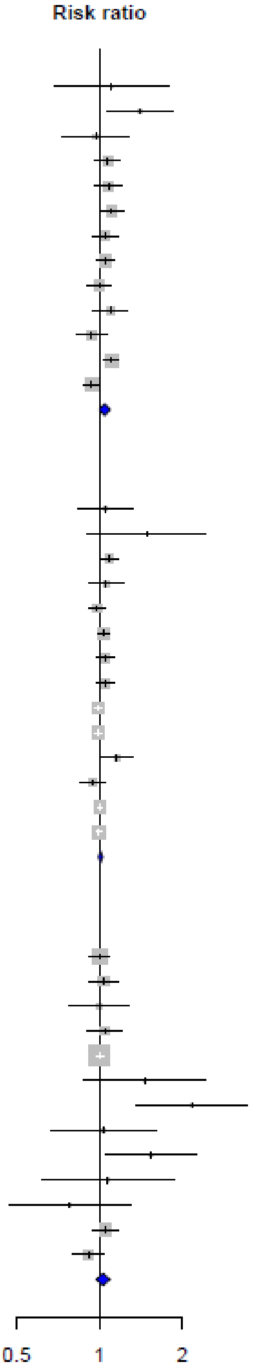

© 2021 Egunsola O et al. *JAMA Network Open.*

eFigure 16. GMT: 3 µg ID vs 15 µg IM

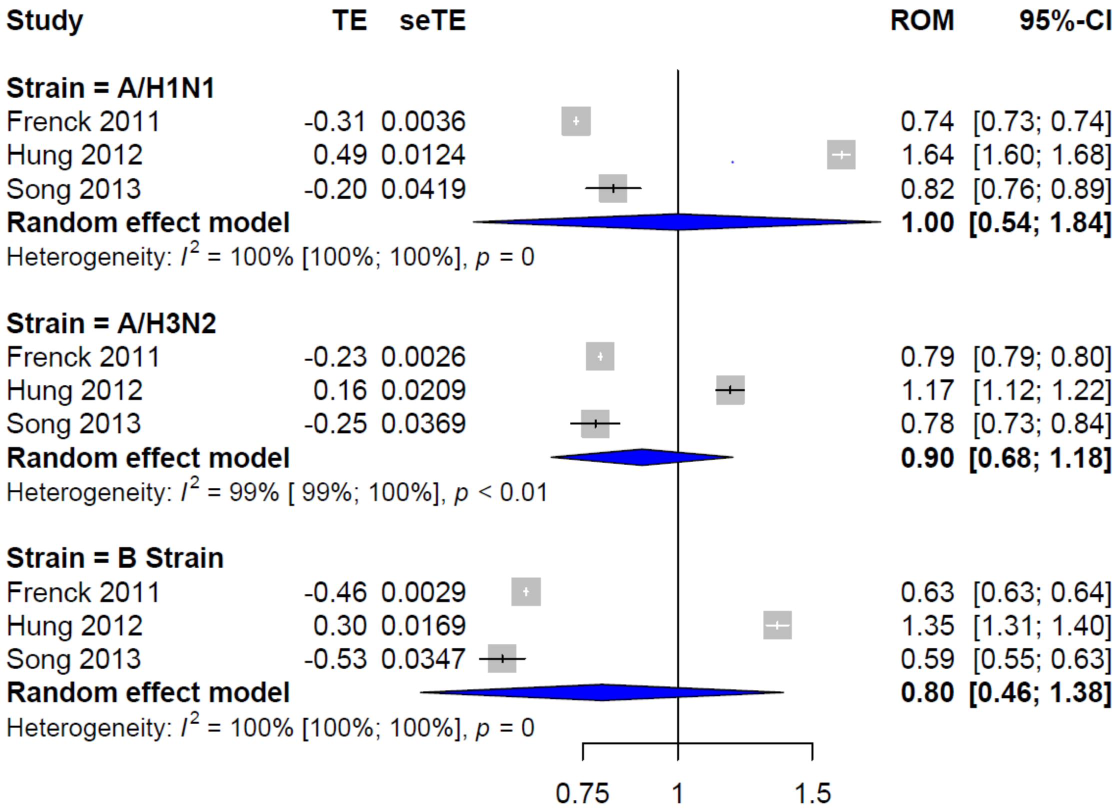

© 2021 Egunsola O et al. *JAMA Network Open.*

eFigure 17. GMT: 6 µg ID vs 15 µg IM

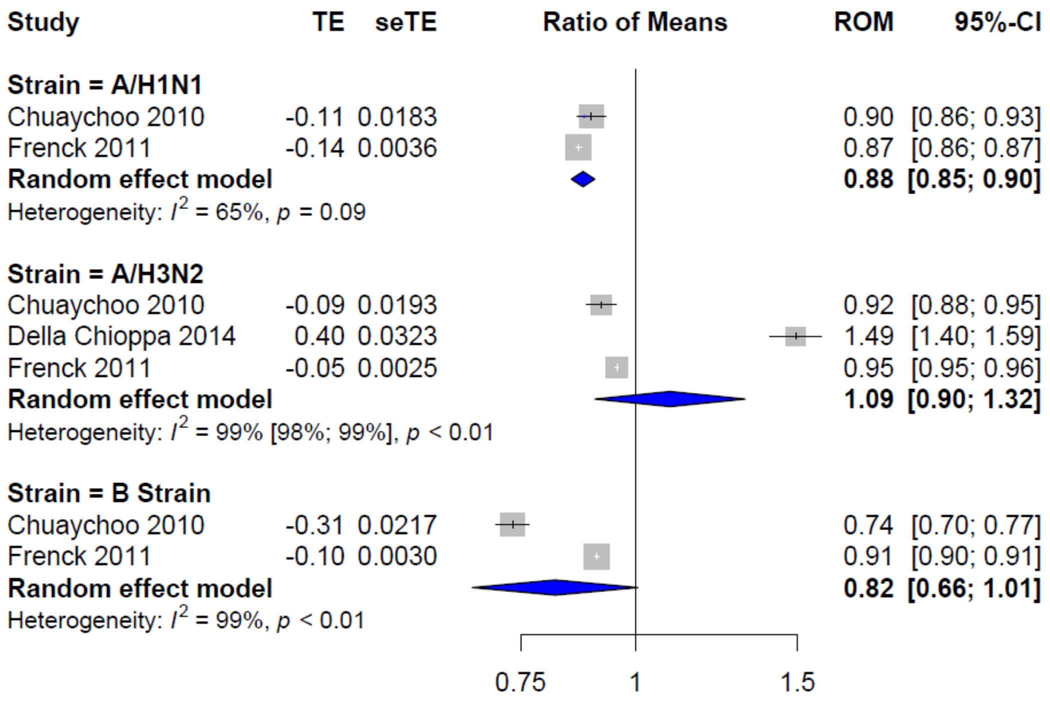

© 2021 Egunsola O et al. *JAMA Network Open.*

eFigure 18. GMT: 9 μg ID vs 15 μg IM
(* Two Doses, †Soluvia Device)

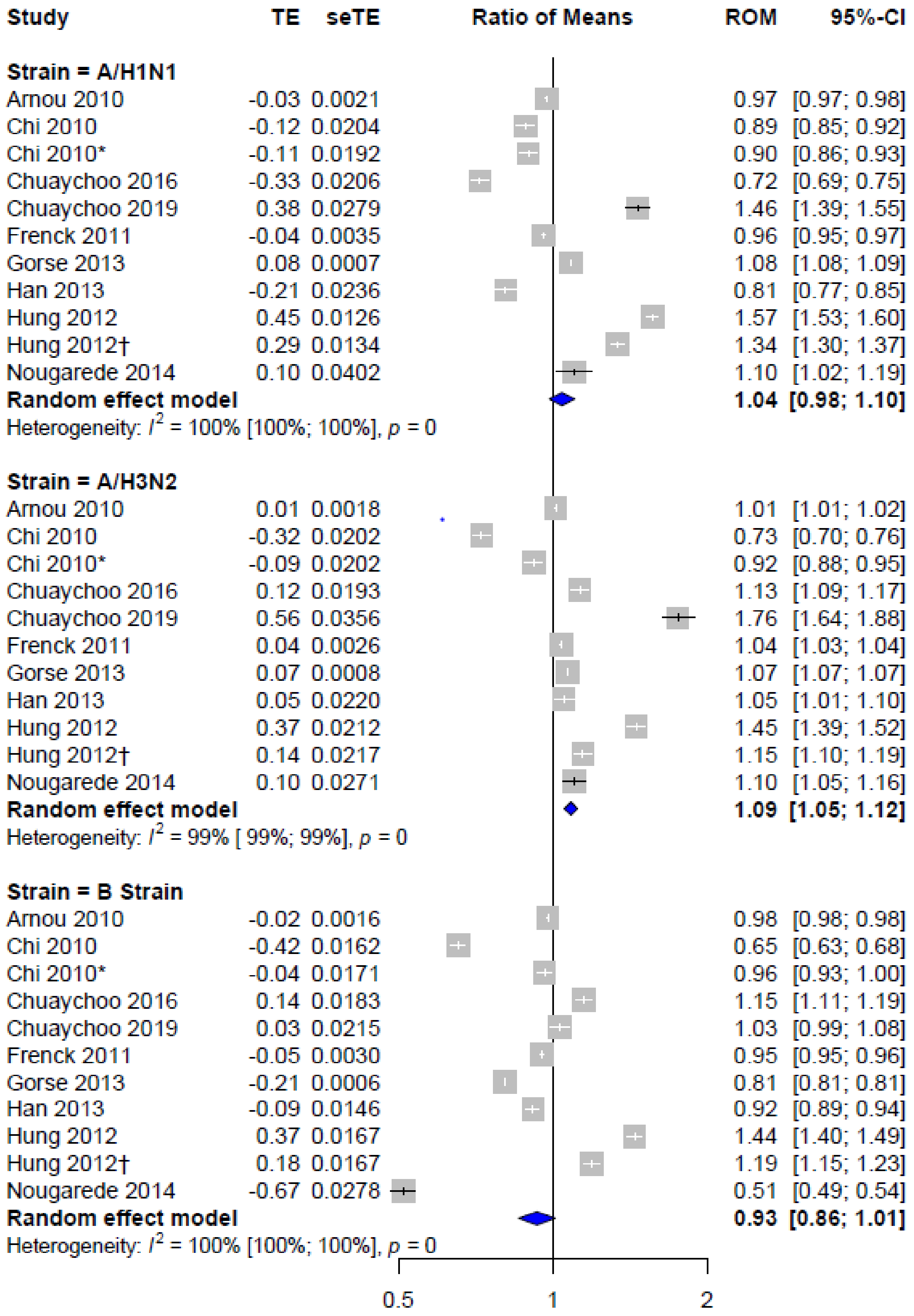

© 2021 Egunsola O et al. *JAMA Network Open.*

eFigure 19. GMT: in Older Adults 9 µg ID vs 15 µg IM

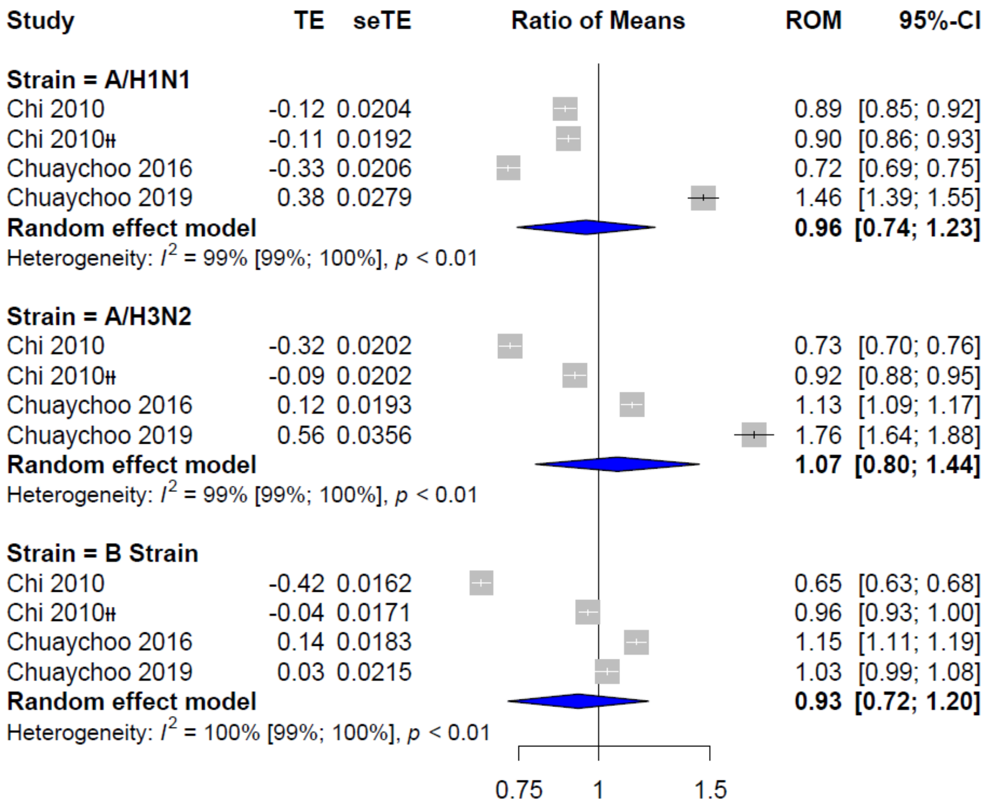

© 2021 Egunsola O et al. *JAMA Network Open.*

eFigure 20. GMT: 15 µg ID vs 15 µg IM

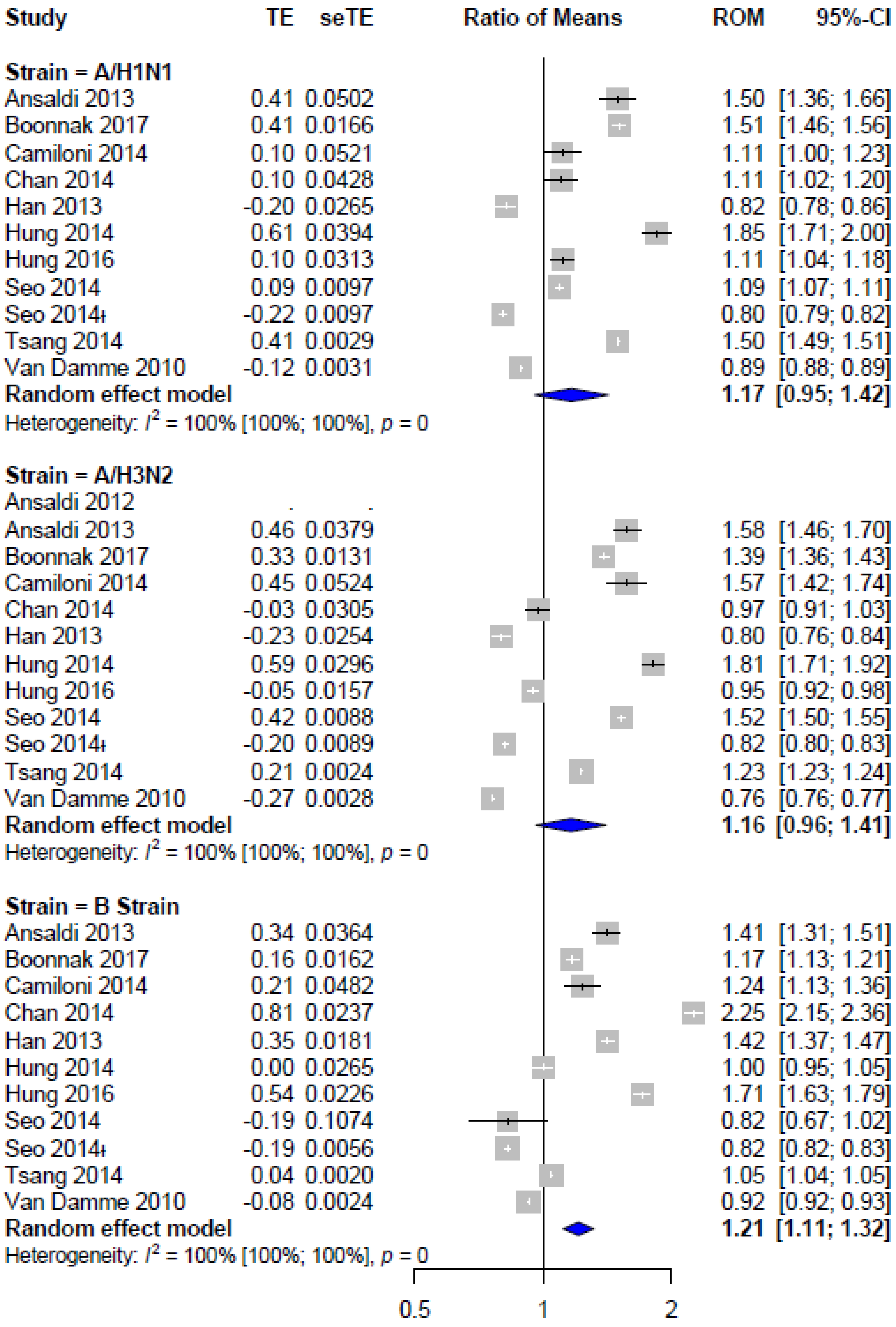

© 2021 Egunsola O et al. *JAMA Network Open.*

eFigure 21. GMT in Older Adults: 15 µg ID vs 15 µg IM
(ɨ Adjuvanted, †≥60 Years Old)

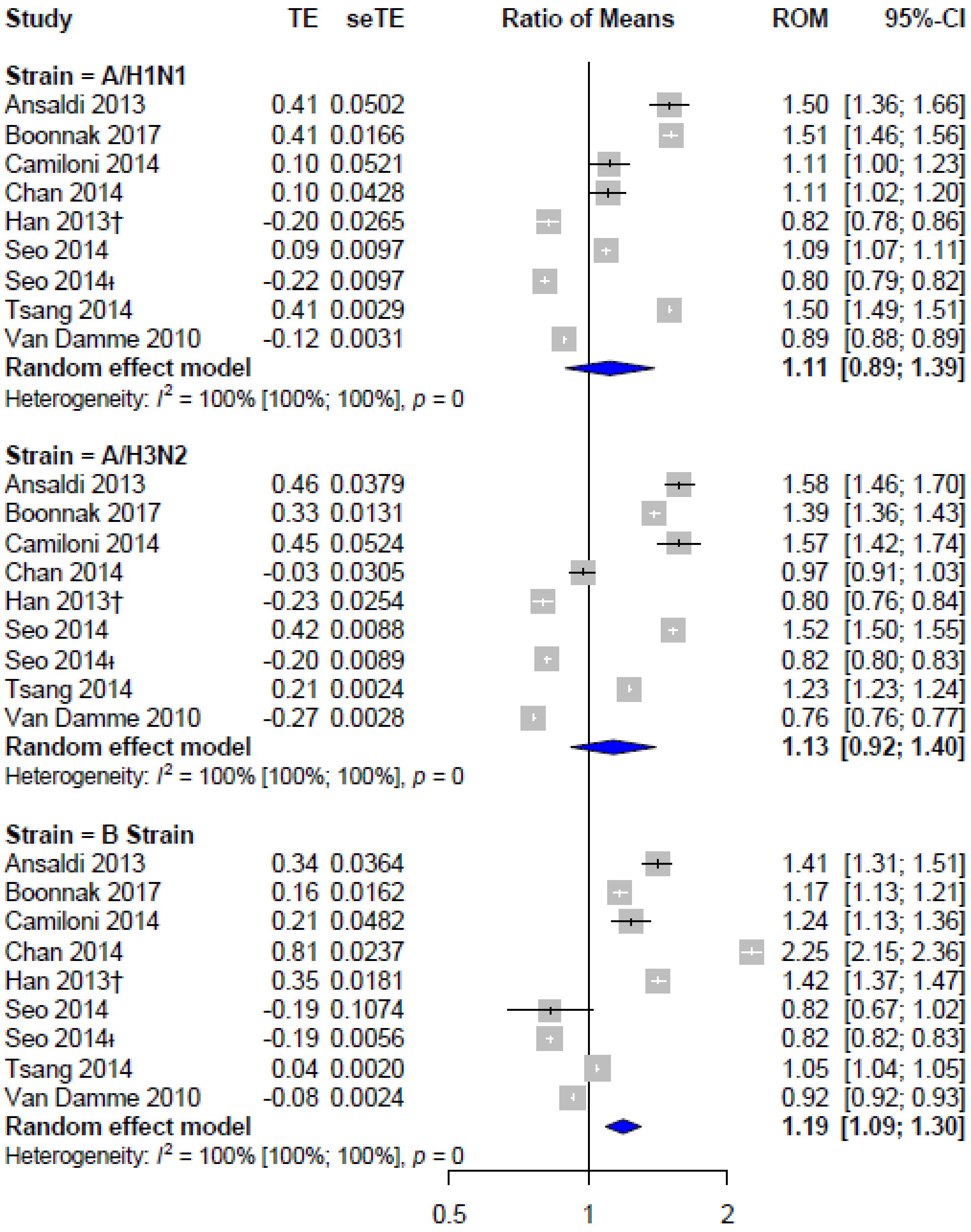

© 2021 Egunsola O et al. *JAMA Network Open.*

eFigure 22. Local Adverse Events: 3 µg ID vs 15 µg IM

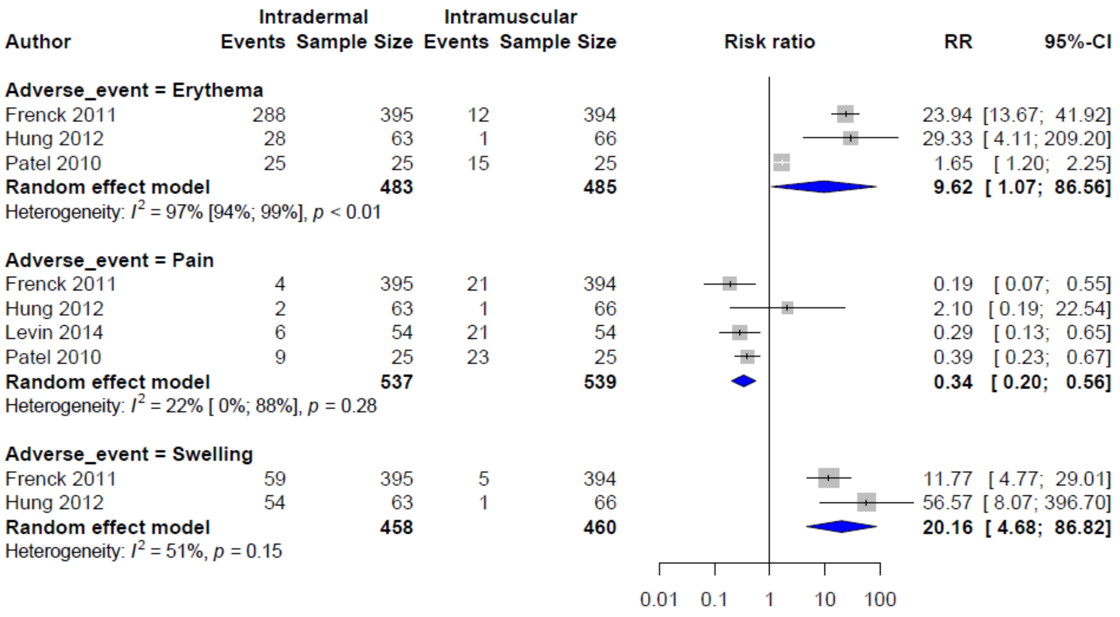

eFigure 23. Local Adverse Events: 6 µg ID vs 15 µg IM

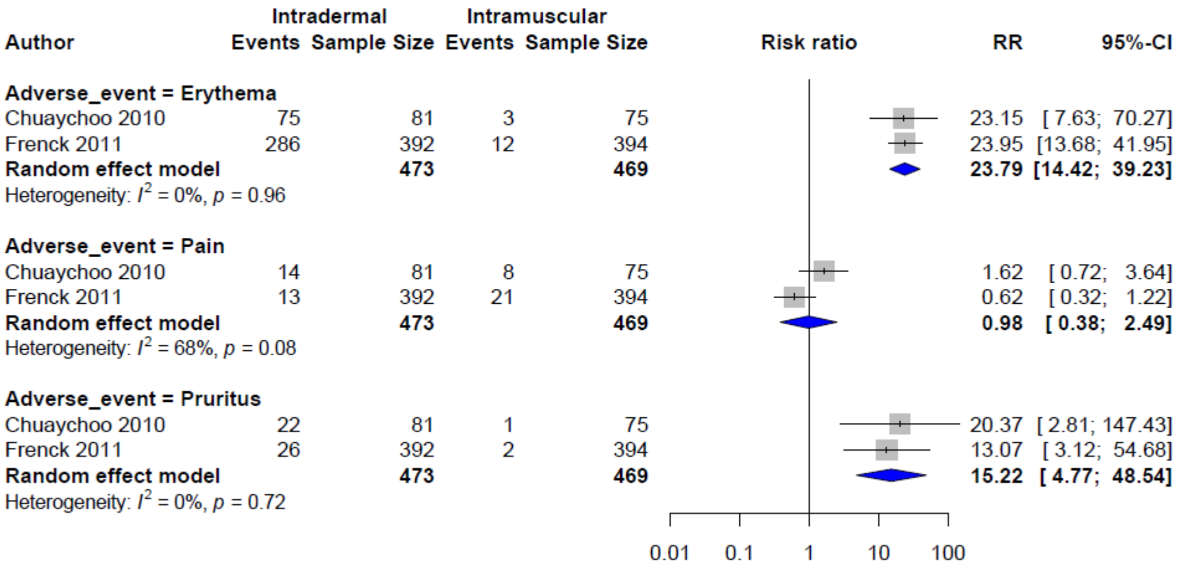

© 2021 Egunsola O et al. *JAMA Network Open.*

eFigure 24. Local Adverse Events: 9 µg ID vs 15 µg IM
(††Two Doses, ‡Soluvia Device)

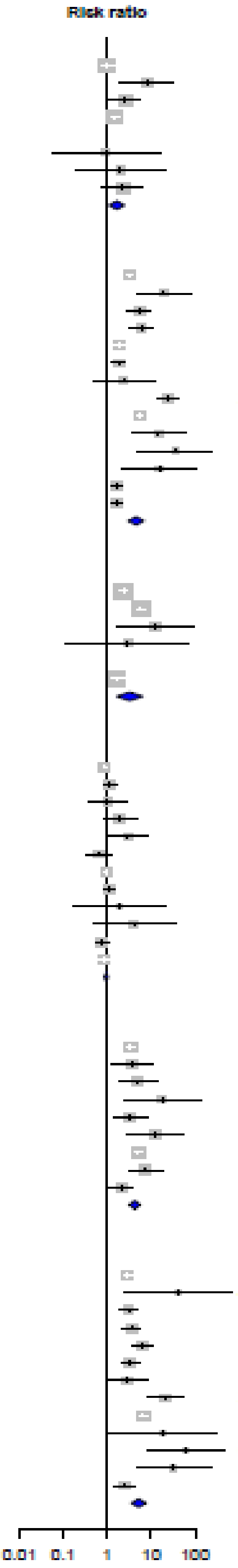

Author	Intradermal Events	Intradermal Sample Size	Intramuscular Events	Intramuscular Sample Size	RR	95%-CI
**Adverse_event = Ecchymosis**						
Arnou 2010	177	1773	44	444	1.01	[0.74; 1.38]
Chuaychoo 2016	16	67	2	67	8.00	[1.91; 33.44]
Chuaychoo 2019	16	36	6	35	2.59	[1.15; 5.86]
Gorse 2013	261	2802	86	1394	1.51	[1.19; 1.91]
Han 2013	0	60	0	60		
Hung 2012	1	68	1	66	0.97	[0.06; 15.20]
Hung 2012‡	2	65	1	66	2.03	[0.19; 21.85]
Nougarede 2014	8	38	4	42	2.21	[0.72; 6.76]
**Random effect model**		**4809**		**2174**	**1.67**	**[1.12; 2.48]**
Heterogeneity: $I^2 = 55\%$ [0%; 81%], $p = 0.04$						
**Adverse_event = Erythema**						
Arnou 2010	1497	1774	113	444	3.32	[2.82; 3.89]
Carter 2019	35	50	2	56	19.60	[4.97; 77.36]
Chi 2010	48	63	9	64	5.42	[2.91; 10.08]
Chi 2010††	56	65	9	64	6.13	[3.32; 11.31]
Chuaychoo 2016	67	67	34	67	1.96	[1.55; 2.47]
Chuaychoo 2019	36	36	19	35	1.82	[1.35; 2.45]
Esposito 2011	5	38	2	37	2.43	[0.50; 11.77]
Frenck 2011	289	390	12	394	24.33	[13.90; 42.60]
Gorse 2013	2141	2802	184	1394	5.79	[5.05; 6.63]
Han 2013	29	60	2	60	14.50	[3.62; 58.07]
Hung 2012	36	68	1	66	34.94	[4.93; 247.54]
Hung 2012‡	15	65	1	66	15.23	[2.07; 111.99]
Nougarede 2014	34	38	23	42	1.63	[1.22; 2.20]
Patel 2010	25	25	15	25	1.65	[1.20; 2.25]
**Random effect model**		**6641**		**2814**	**4.68**	**[3.06; 6.82]**
Heterogeneity: $I^2 = 94\%$ [91%; 96%], $p < 0.01$						
**Adverse_event = Induration**						
Arnou 2010	1078	1773	116	444	2.33	[1.98; 2.73]
Gorse 2013	1636	2802	139	1394	5.86	[4.99; 6.88]
Han 2013	13	60	1	60	13.00	[1.76; 96.27]
Hung 2012	1	68	0	66	2.91	[0.12; 70.23]
Hung 2012‡	0	65	0	66		
Nougarede 2014	34	38	22	42	1.71	[1.25; 2.32]
**Random effect model**		**4806**		**2072**	**3.27**	**[1.66; 6.46]**
Heterogeneity: $I^2 = 95\%$ [92%; 97%], $p < 0.01$						
**Adverse_event = Pain**						
Arnou 2010	765	1774	215	444	0.89	[0.80; 0.99]
Carter 2019	31	50	30	56	1.16	[0.84; 1.60]
Chi 2010	7	63	7	64	1.02	[0.38; 2.73]
Chi 2010††	14	65	7	64	1.97	[0.85; 4.56]
Esposito 2011	12	38	4	37	2.92	[1.04; 8.24]
Frenck 2011	14	390	21	394	0.67	[0.35; 1.31]
Gorse 2013	1429	2802	749	1394	0.95	[0.89; 1.01]
Han 2013	36	60	32	60	1.12	[0.82; 1.54]
Hung 2012	2	68	1	66	1.94	[0.18; 20.90]
Hung 2012‡	4	65	1	66	4.06	[0.47; 35.37]
Nougarede 2014	22	38	31	42	0.78	[0.57; 1.09]
Patel 2010	19	25	23	25	0.83	[0.64; 1.06]
**Random effect model**		**6438**		**2712**	**0.96**	**[0.86; 1.06]**
Heterogeneity: $I^2 = 34\%$ [0%; 67%], $p = 0.12$						
**Adverse_event = Pruritus**						
Arnou 2010	795	1774	58	444	3.43	[2.68; 4.39]
Chi 2010	15	63	4	64	3.81	[1.34; 10.85]
Chi 2010††	21	65	4	64	5.17	[1.88; 14.22]
Chuaychoo 2016	18	67	1	67	18.00	[2.47; 131.02]
Chuaychoo 2019	17	36	5	35	3.31	[1.37; 7.98]
Frenck 2011	24	390	2	394	12.12	[2.88; 50.95]
Gorse 2013	1314	2802	130	1394	5.03	[4.25; 5.95]
Han 2013	37	60	5	60	7.40	[3.12; 17.53]
Nougarede 2014	21	38	11	42	2.11	[1.18; 3.78]
**Random effect model**		**6296**		**2684**	**4.24**	**[3.16; 5.70]**
Heterogeneity: $I^2 = 58\%$ [8%; 79%], $p = 0.02$						
**Adverse_event = Swelling**						
Arnou 2010	1097	1773	92	444	2.99	[2.48; 3.59]
Carter 2019	18	50	0	56	41.40	[2.56; 669.44]
Chi 2010	39	63	13	64	3.05	[1.81; 5.14]
Chi 2010††	47	65	13	64	3.56	[2.14; 5.92]
Chuaychoo 2016	66	67	11	67	6.00	[3.49; 10.31]
Chuaychoo 2019	36	36	10	35	3.38	[2.04; 5.62]
Esposito 2011	12	38	4	37	2.92	[1.04; 8.24]
Frenck 2011	105	390	5	394	21.22	[8.75; 51.46]
Gorse 2013	1592	2802	117	1394	6.77	[5.67; 8.08]
Han 2013	9	60	0	60	19.00	[1.13; 319.21]
Hung 2012	64	68	1	66	62.12	[8.87; 434.85]
Hung 2012‡	32	65	1	66	32.49	[4.57; 230.83]
Nougarede 2014	26	38	12	42	2.39	[1.42; 4.05]
**Random effect model**		**6616**		**2789**	**6.23**	**[3.68; 7.82]**
Heterogeneity: $I^2 = 84\%$ [75%; 90%], $p < 0.01$						

© 2021 Egunsola O et al. *JAMA Network Open.*

eFigure 25. Local Adverse Events: 15 μg ID vs 15 μg IM
(*Inflexa vs Inflexa, ** Intanza vs Inflexa,†Inflexa vs Fluad, ‡Intanza vs Fluad, ǂ
Adjuvanted)

Author	Intradermal Events	Intradermal Sample Size	Intramuscular Events	Intramuscular Sample Size	RR	95%-CI
**Adverse_event = Ecchymosis**						
Ansaldi 2013	2	28	1	27	1.93	[0.19; 20.05]
Boonnak 2017	3	111	1	110	2.97	[0.31; 28.14]
Chan 2014	0	50	0	50		
Han 2013	1	60	1	60	1.00	[0.06; 15.62]
Hung 2014	0	31	0	30		
Levin 2016*	1	61	4	63	0.26	[0.03; 2.25]
Levin 2016**	0	60	4	63	0.12	[0.01; 2.12]
Levin 2016‡	1	61	1	63	1.03	[0.07; 16.15]
Levin 2016ǂ	0	60	1	63	0.35	[0.01; 8.42]
Tsang 2014	36	635	19	319	0.95	[0.56; 1.63]
Van Damme 2010	20	398	13	397	1.53	[0.77; 3.04]
**Random effect model**		**1666**		**1246**	**1.07**	**[0.72; 1.67]**
Heterogeneity: I^2 = 0% [0%; 58%], p = 0.57						
**Adverse_event = Erythema**						
Ansaldi 2013	9	28	2	27	4.34	[1.03; 18.28]
Boonnak 2017	39	111	2	110	19.32	[4.78; 78.08]
Chan 2014	13	50	2	50	6.50	[1.55; 27.33]
Esposito 2011	4	37	2	37	2.00	[0.39; 10.26]
Garg 2015	20	40	0	40	41.00	[2.57; 655.21]
Han 2013	25	60	6	60	4.17	[1.84; 9.42]
Hung 2014	17	31	5	30	3.29	[1.39; 7.79]
Hung 2016	3	40	1	40	3.00	[0.33; 27.63]
Levin 2016*	56	61	19	63	3.04	[2.08; 4.47]
Levin 2016**	52	60	19	63	2.87	[1.95; 4.24]
Levin 2016‡	56	61	15	63	3.86	[2.46; 6.04]
Levin 2016ǂ	52	60	15	63	3.64	[2.31; 5.72]
Seo 2014	11	111	4	113	2.80	[0.92; 8.53]
Seo 2014†	11	111	6	111	1.83	[0.70; 4.78]
Tsang 2014	425	635	48	319	4.45	[3.41; 5.81]
Van Damme 2010	245	398	66	397	3.70	[2.93; 4.68]
**Random effect model**		**1884**		**1688**	**3.68**	**[3.18; 4.26]**
Heterogeneity: I^2 = 9% [0%; 45%], p = 0.35						
**Adverse_event = Induration**						
Ansaldi 2013	7	28	2	27	3.38	[0.77; 14.82]
Chan 2014	0	50	0	50		
Han 2013	6	60	3	60	2.00	[0.52; 7.63]
Hung 2014	9	31	8	30	1.09	[0.48; 2.45]
Levin 2016*	34	61	12	63	2.93	[1.68; 5.10]
Levin 2016**	31	60	12	63	2.71	[1.54; 4.77]
Levin 2016‡	34	61	11	63	3.19	[1.78; 5.71]
Levin 2016ǂ	31	60	11	63	2.96	[1.64; 5.34]
Tsang 2014	309	635	32	319	4.85	[3.46; 6.80]
Van Damme 2010	135	398	46	397	2.93	[2.16; 3.97]
**Random effect model**		**1444**		**1136**	**2.98**	**[2.32; 3.84]**
Heterogeneity: I^2 = 43% [0%; 73%], p = 0.08						
**Adverse_event = Pain**						
Ansaldi 2013	4	28	3	27	1.29	[0.32; 5.22]
Boonnak 2017	21	111	19	110	1.10	[0.62; 1.92]
Chan 2014	2	50	3	50	0.67	[0.12; 3.82]
Esposito 2011	11	37	4	37	2.75	[0.96; 7.86]
Garg 2015	5	40	14	40	0.36	[0.14; 0.90]
Han 2013	19	60	19	60	1.00	[0.59; 1.69]
Hung 2014	13	31	4	30	3.15	[1.15; 8.57]
Hung 2016	1	40	2	40	0.50	[0.05; 5.30]
Levin 2016*	11	61	21	63	0.54	[0.29; 1.02]
Levin 2016**	22	60	21	63	1.10	[0.68; 1.78]
Levin 2016‡	11	61	28	63	0.41	[0.22; 0.74]
Levin 2016ǂ	22	60	28	63	0.82	[0.54; 1.27]
Seo 2014	7	111	8	113	0.89	[0.33; 2.37]
Seo 2014†	7	111	12	111	0.58	[0.24; 1.43]
Tsang 2014	173	635	56	319	1.55	[1.19; 2.03]
Van Damme 2010	80	398	82	397	0.97	[0.74; 1.28]
**Random effect model**		**1884**		**1688**	**0.94**	**[0.72; 1.21]**
Heterogeneity: I^2 = 61% [33%; 78%], p < 0.01						
**Adverse_event = Pruritus**						
Ansaldi 2013	6	28	1	27	5.79	[0.74; 44.94]
Boonnak 2017	11	111	3	110	3.63	[1.04; 12.67]
Garg 2015	1	40	2	40	0.50	[0.05; 5.30]
Han 2013	34	60	7	60	4.86	[2.34; 10.08]
Tsang 2014	201	635	27	319	3.74	[2.56; 5.46]
Van Damme 2010	113	398	26	397	4.34	[2.90; 6.49]
**Random effect model**		**1272**		**963**	**4.01**	**[3.13; 5.16]**
Heterogeneity: I^2 = 0% [0%; 65%], p = 0.60						
**Adverse_event = Swelling**						
Ansaldi 2013	5	28	1	27	4.82	[0.60; 38.63]
Boonnak 2017	50	111	11	110	4.50	[2.48; 8.18]
Chan 2014	10	50	1	50	10.00	[1.33; 75.23]
Esposito 2011	11	37	4	37	2.75	[0.96; 7.86]
Garg 2015	29	40	3	40	9.67	[3.20; 29.18]
Han 2013	11	60	2	60	5.50	[1.27; 23.77]
Hung 2014	12	31	14	30	0.83	[0.46; 1.49]
Hung 2016	8	40	3	40	2.67	[0.76; 9.33]
Seo 2014	7	111	4	113	1.78	[0.54; 5.92]
Seo 2014†	7	111	3	111	2.33	[0.62; 8.79]
Tsang 2014	283	635	22	319	6.46	[4.28; 9.76]
Van Damme 2010	148	398	40	397	3.69	[2.68; 5.08]
**Random effect model**		**1862**		**1334**	**3.47**	**[2.20; 5.46]**
Heterogeneity: I^2 = 72% [50%; 84%], p < 0.01						

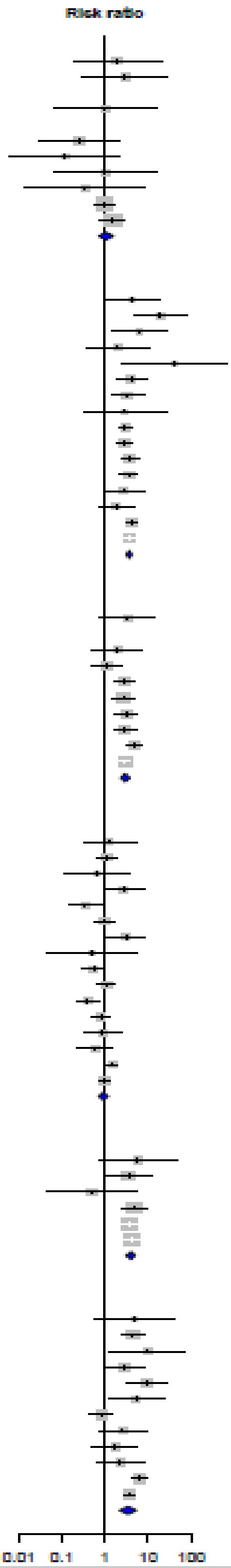

© 2021 Egunsola O et al. *JAMA Network Open.*

eFigure 26. Systemic Adverse Events: 3 μg ID vs 15 μg IM

Author	Intradermal Events	Intradermal Sample Size	Intramuscular Events	Intramuscular Sample Size	RR	95%-CI
**Adverse_event = Headache**						
Frenck 2011	107	395	99	394	1.08	[0.85; 1.36]
Hung 2012	6	63	5	66	1.26	[0.40; 3.91]
**Random effect model**		**458**		**460**	**1.08**	**[0.86; 1.37]**
Heterogeneity: I^2 = 0%, p = 0.80						

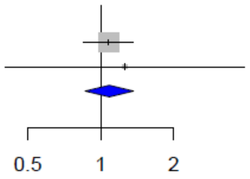

eFigure 27. Systemic Adverse Events: 6 μg ID vs 15 μg IM

Author	Intradermal Events	Intradermal Sample Size	Intramuscular Events	Intramuscular Sample Size	RR	95%-CI
**Adverse_event = Fever**						
Chuaychoo 2010	3	81	9	75	0.31	[0.09; 1.10]
Frenck 2011	4	392	4	394	1.01	[0.25; 3.99]
**Random effect model**		**473**		**469**	**0.54**	**[0.17; 1.71]**
Heterogeneity: I^2 = 34%, p = 0.22						
**Adverse_event = Headache**						
Chuaychoo 2010	7	81	13	75	0.50	[0.21; 1.18]
Frenck 2011	110	392	99	394	1.12	[0.88; 1.41]
**Random effect model**		**473**		**469**	**0.83**	**[0.39; 1.78]**
Heterogeneity: I^2 = 68%, p = 0.08						

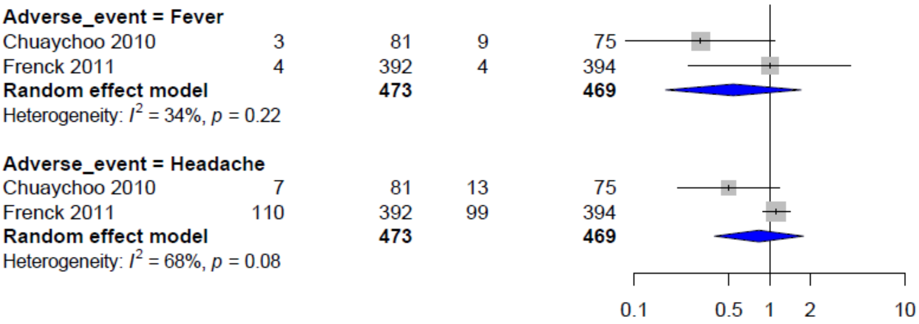

© 2021 Egunsola O et al. *JAMA Network Open.*

eFigure 28. Systemic Adverse Events: 9 µg ID vs 15 µg IM
(‡ Soluvia, ††Two Doses)

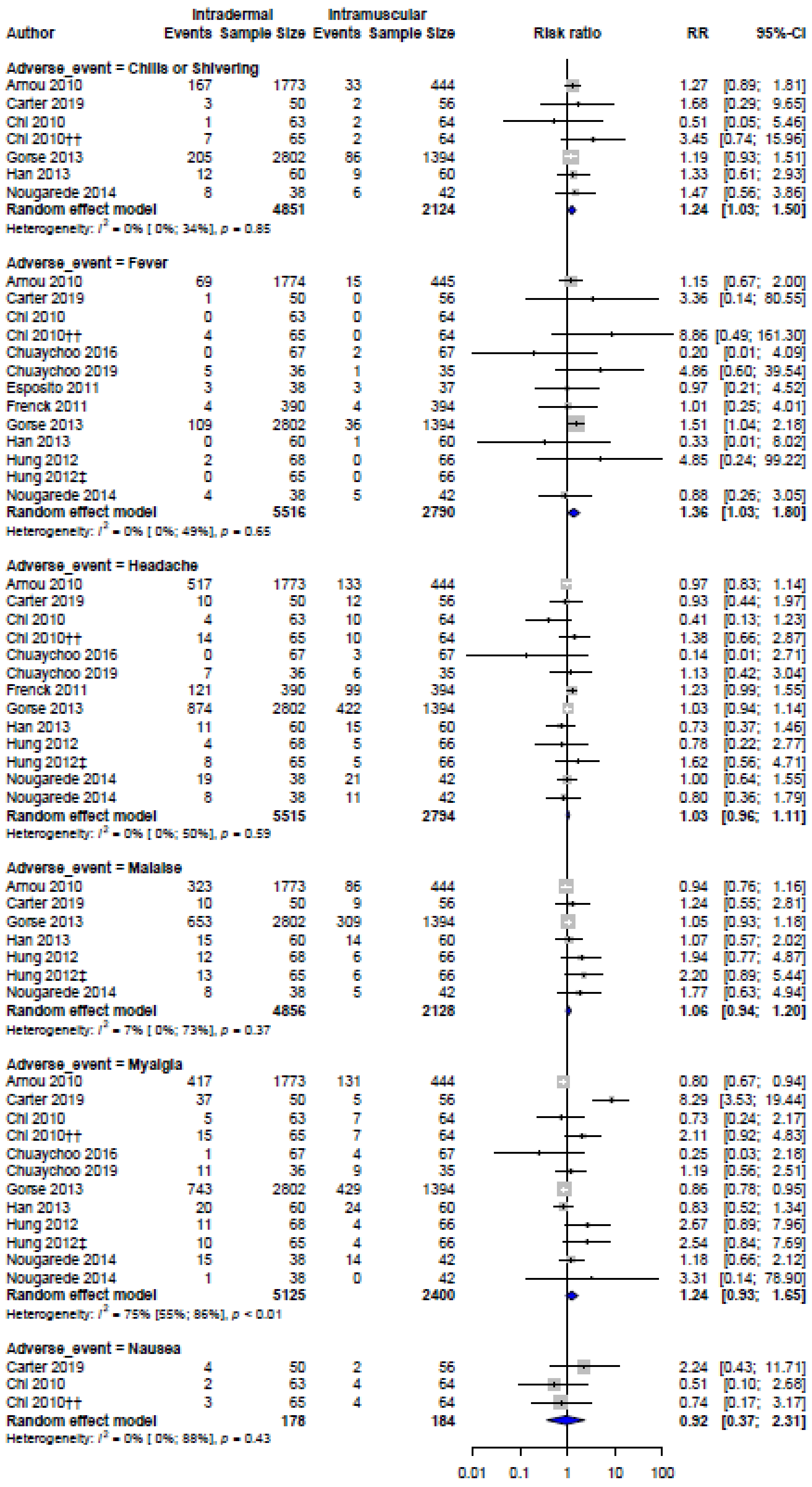

© 2021 Egunsola O et al. *JAMA Network Open.*

eFigure 29. Systemic Adverse Events: 15 µg ID vs 15 µg IM
(*Inflexa vs Inflexa, ** Intanza vs Inflexa,†Inflexa vs Fluad, ‡Intanza vs Fluad, ɫ
Adjuvanted)

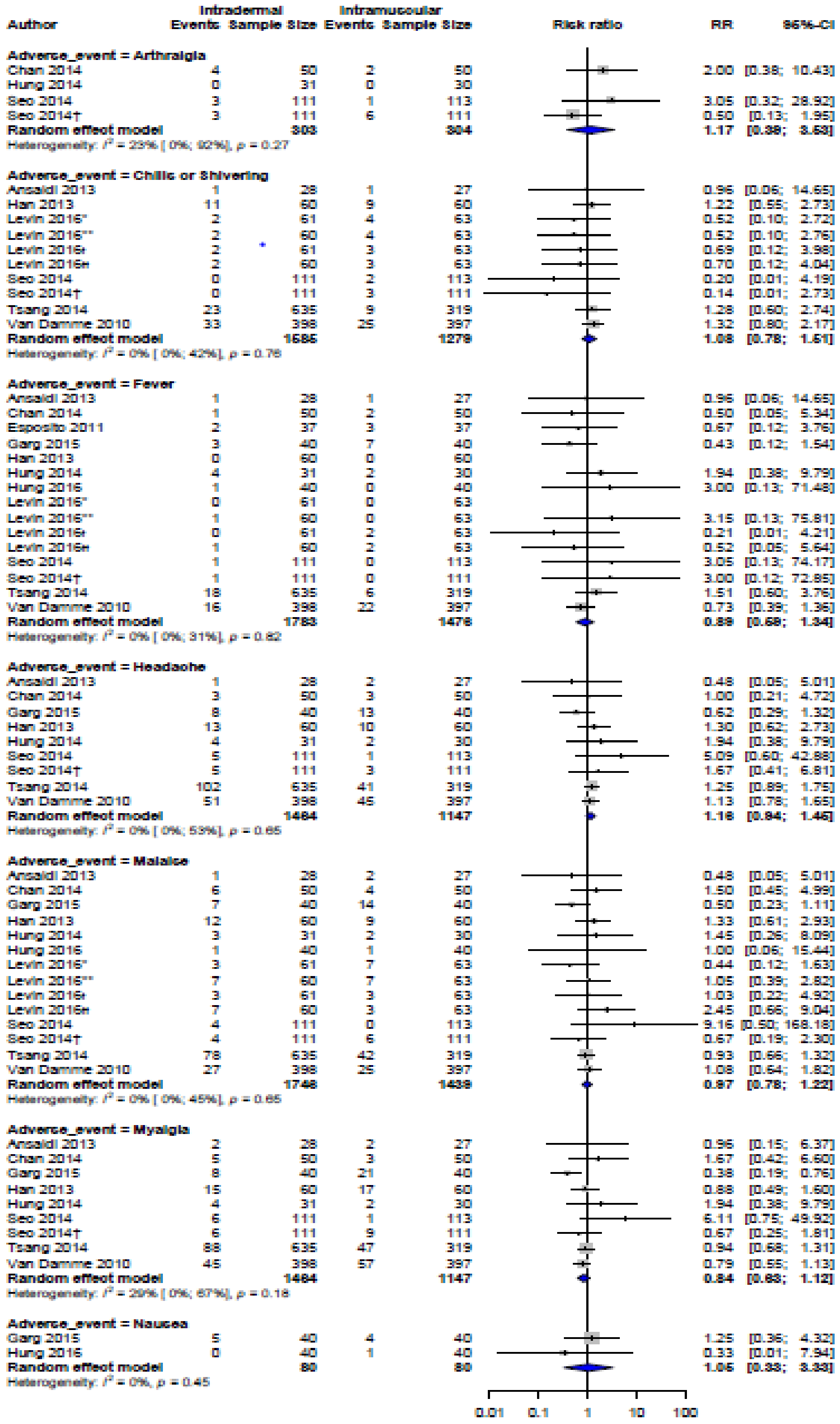

© 2021 Egunsola O et al. *JAMA Network Open.*

eFigure 30. Funnel Plot of Seroconversion: 9 µg ID vs 15 µg IM

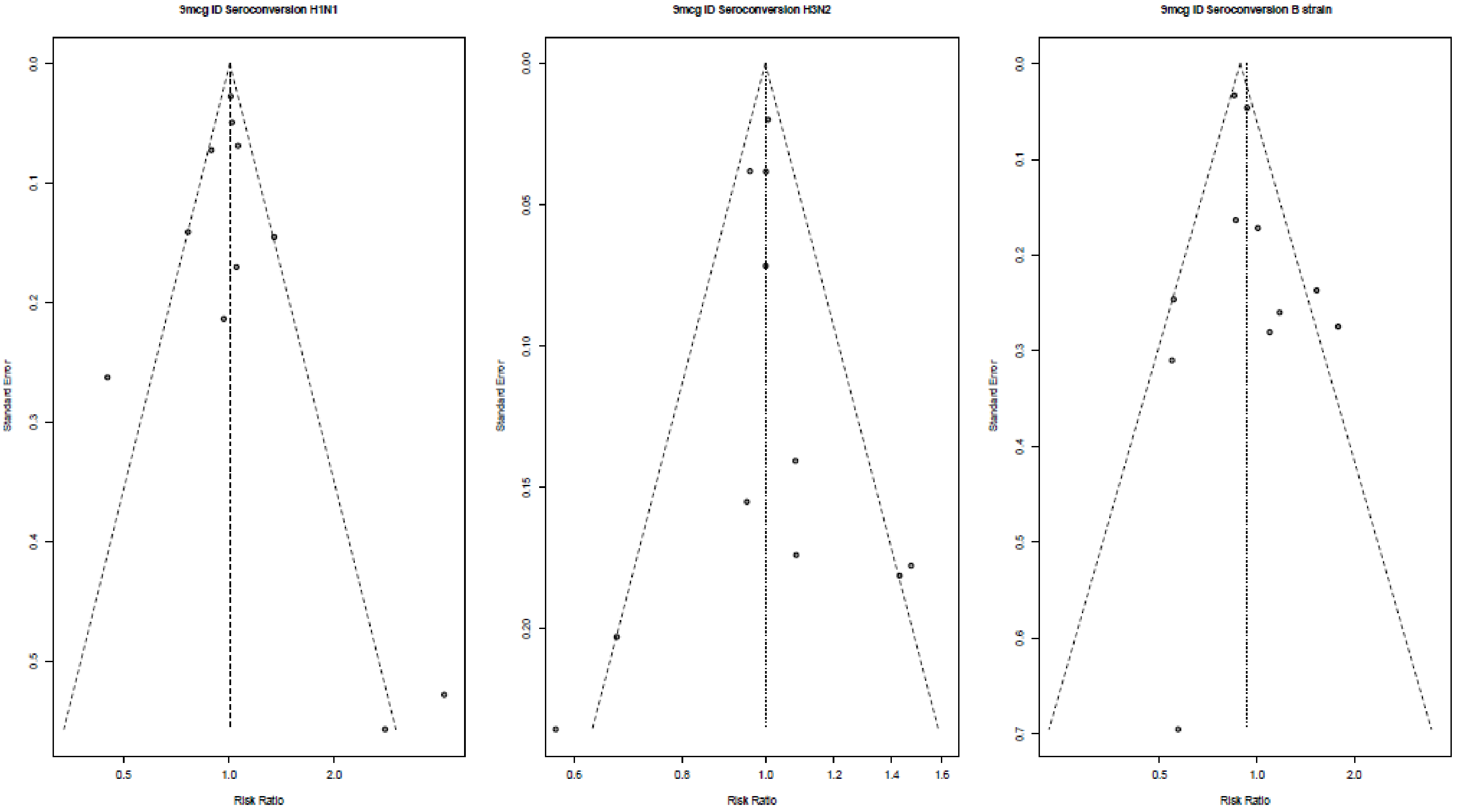

© 2021 Egunsola O et al. *JAMA Network Open.*

eFigure 31. Funnel Plot of Seroprotection: 9 µg vs 15 µg

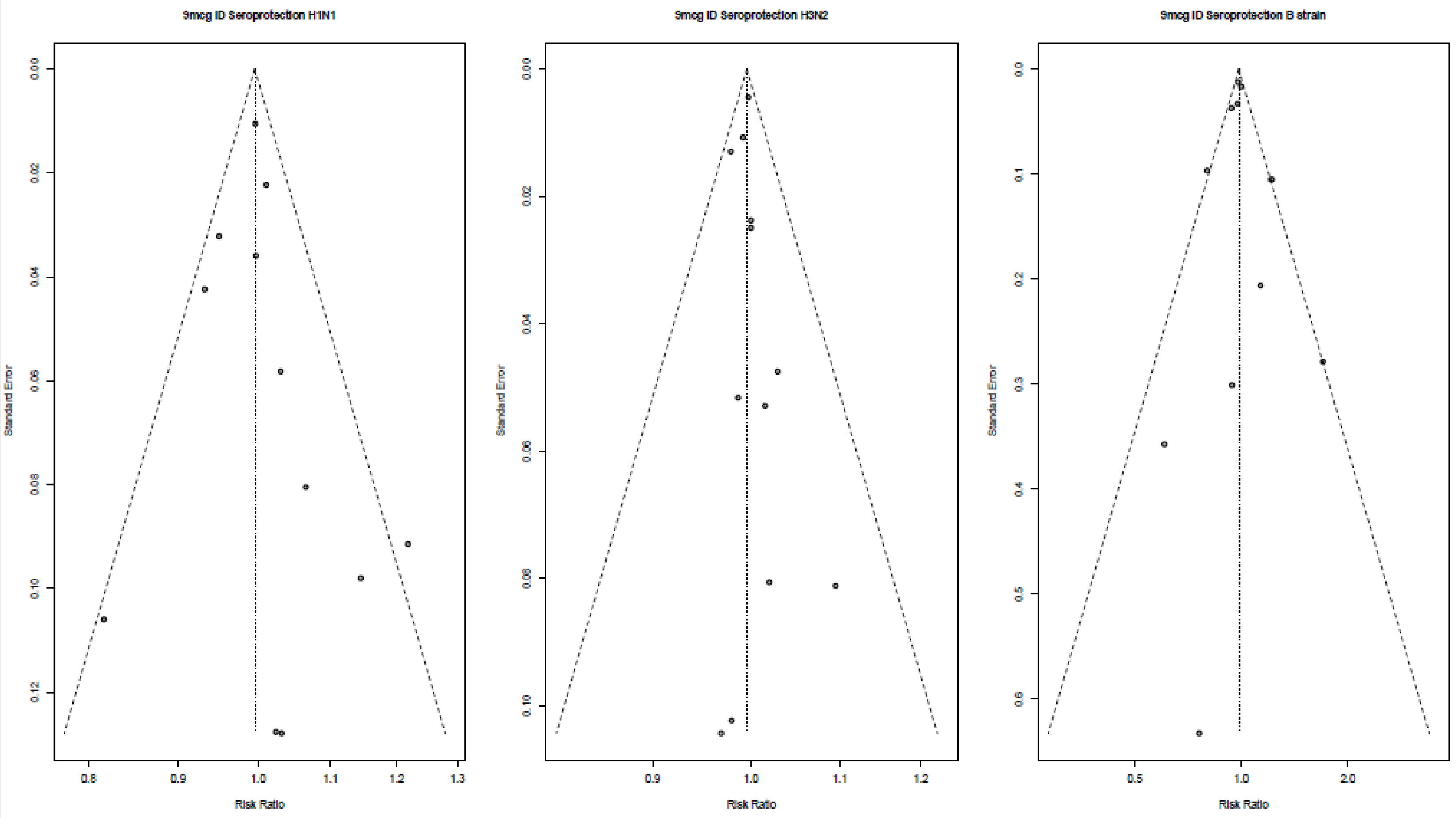

© 2021 Egunsola O et al. *JAMA Network Open.*

eFigure 32. Funnel Plot of GMT: 9 µg ID vs 15 µg IM

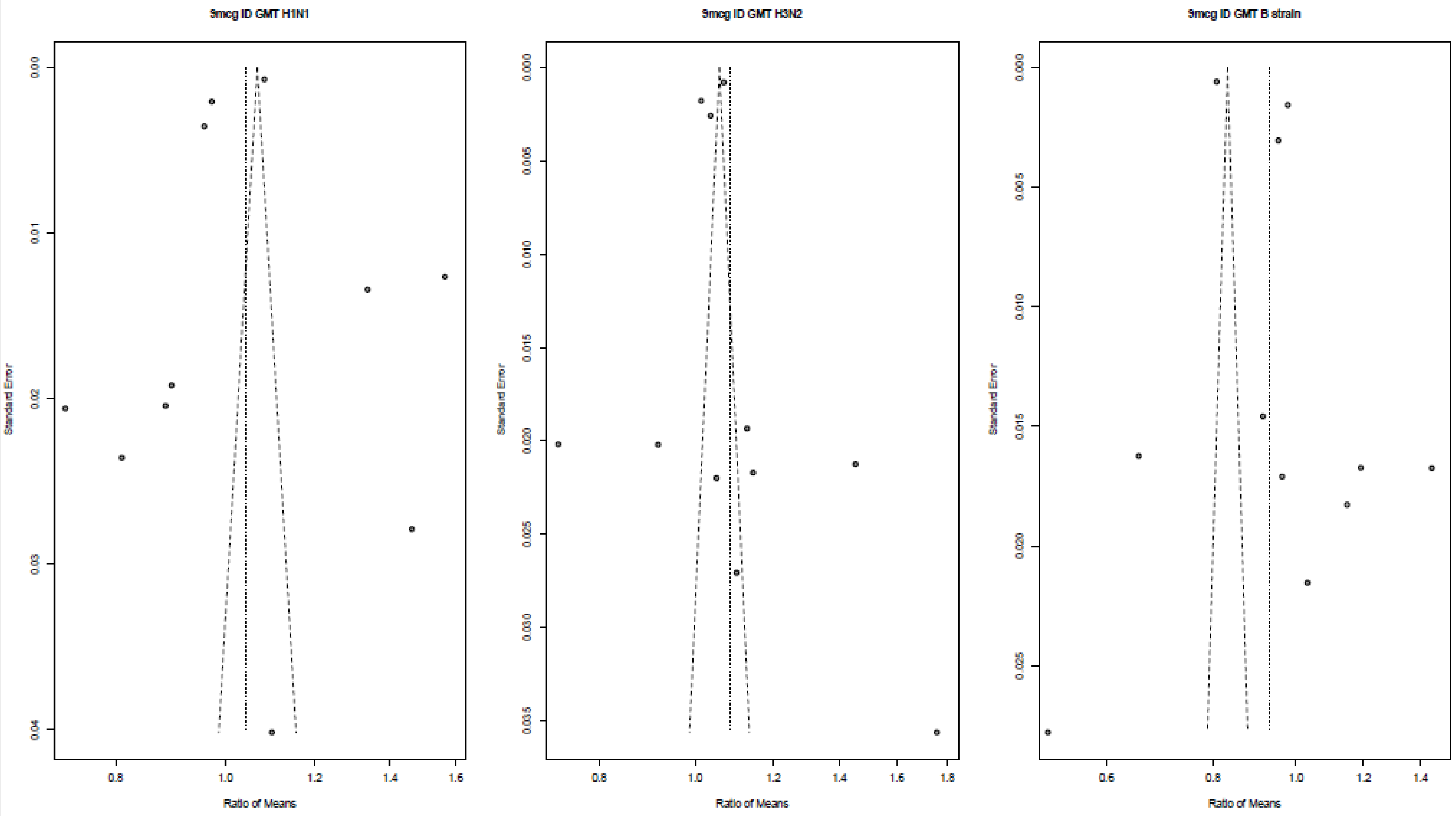

© 2021 Egunsola O et al. *JAMA Network Open.*

eFigure 33. Funnel Plot of Seroconversion: 15 µg ID vs 15 µg IM

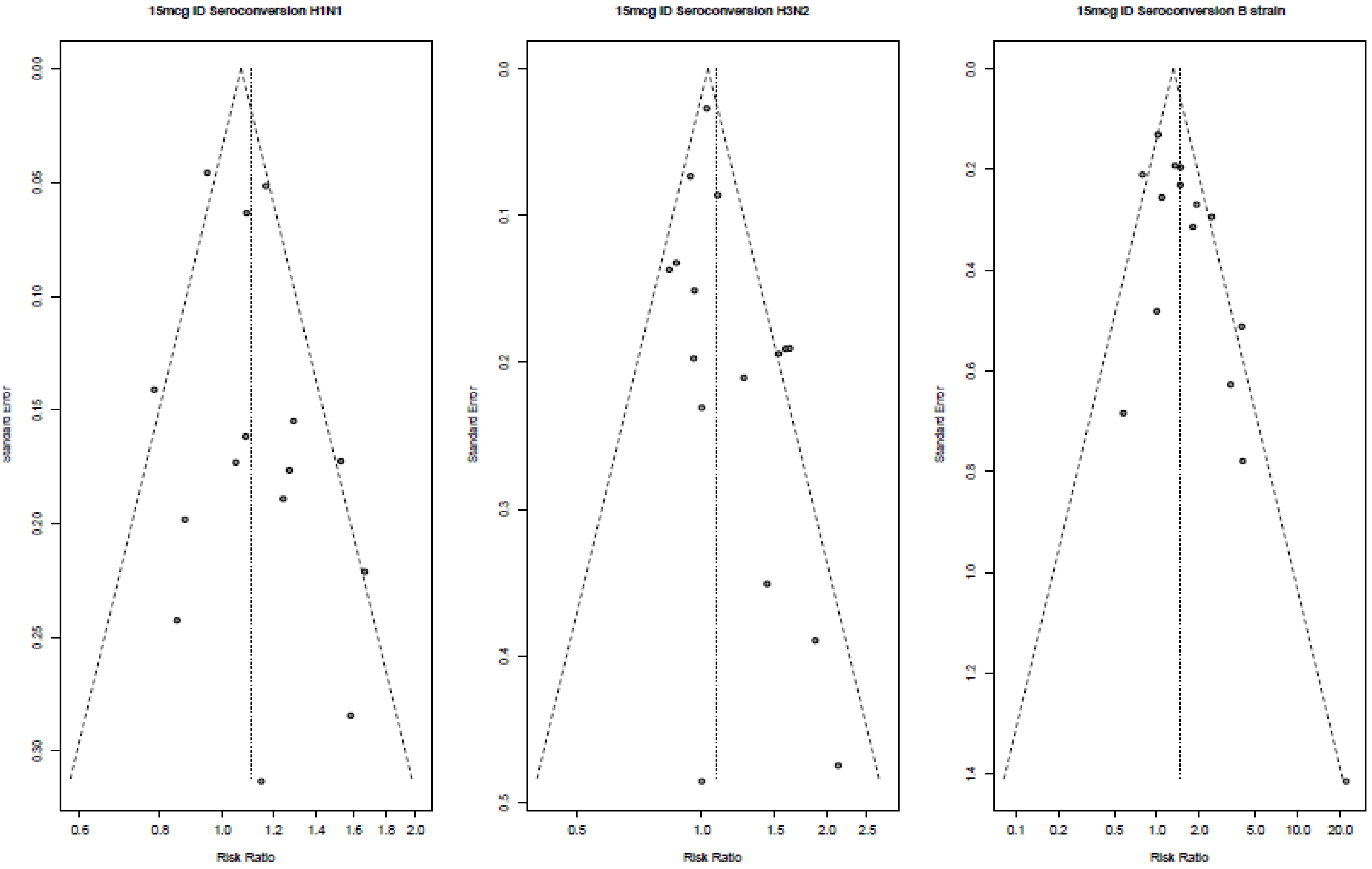

© 2021 Egunsola O et al. *JAMA Network Open.*

eFigure 34. Funnel Plot of Seroprotection: 15 µg ID vs 15 µg IM

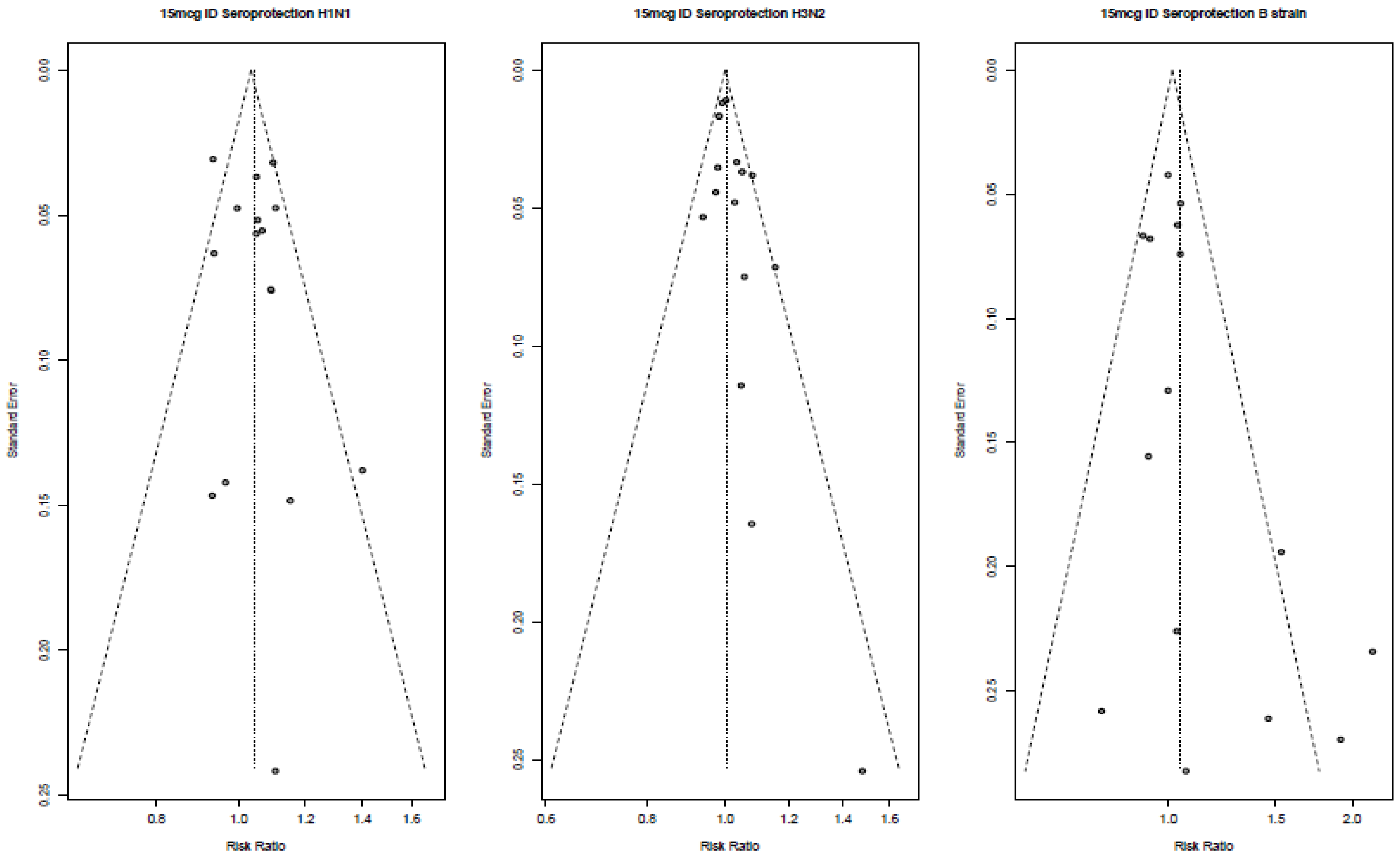

© 2021 Egunsola O et al. *JAMA Network Open.*

eFigure 35. Funnel Plot of GMT: 15 µg ID vs 15 µg IM

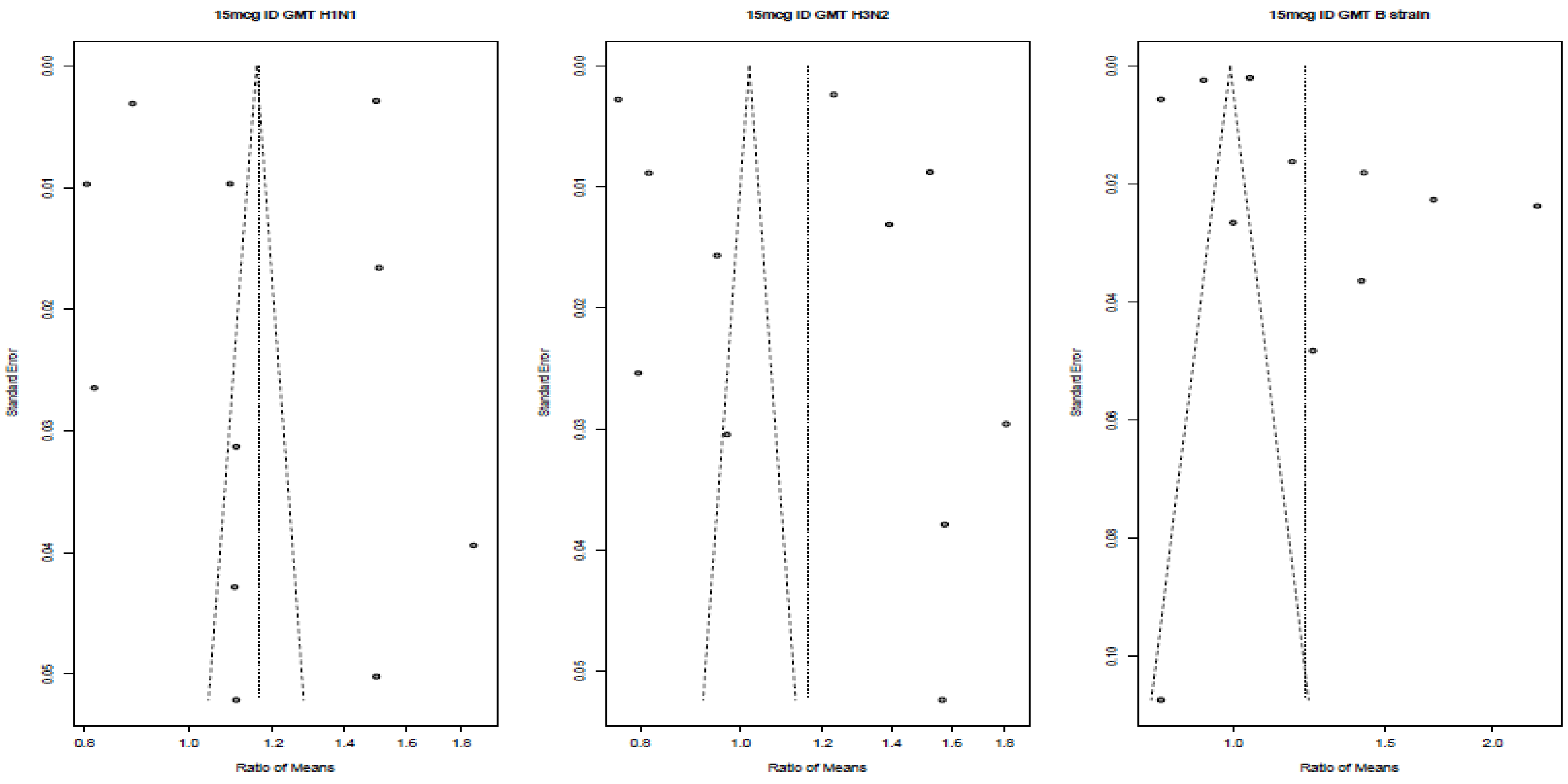

© 2021 Egunsola O et al. *JAMA Network Open.*
